# Photoacclimation of cryptophytes and diatoms to light variability in the Western Antarctic Peninsula

**DOI:** 10.1111/jpy.70158

**Published:** 2026-04-09

**Authors:** Afonso Ferreira, Raul R. Costa, Bruno Jesus, Ana C. Brito, Savênia B. Silveira, Vanda Brotas, Catarina V. Guerreiro, Eduardo Resende Secchi, Carlos Rafael B. Mendes

**Affiliations:** ^1^ MARE – Marine and Environmental Sciences Centre/ARNET – Aquatic Research Network, Faculdade de Ciências Universidade de Lisboa Lisbon Portugal; ^2^ Laboratório de Fitoplâncton e Microorganismos Marinhos Universidade Federal Do Rio Grande‐FURG Rio Grande RS Brazil; ^3^ Departamento de Biologia, Faculdade de Ciências Universidade de Lisboa Lisbon Portugal; ^4^ Laboratório de Estudos dos Oceanos e Clima Universidade Federal do Rio Grande‐FURG Rio Grande RS Brazil; ^5^ Nantes Université Institut des Substances et Organismes de la Mer, ISOMer, UR2160 Nantes France; ^6^ Plymouth Marine Laboratory Prospect Place Plymouth UK; ^7^ Programa de Pós‐graduação em Oceanografia Biológica Universidade Federal do Rio Grande‐FURG Rio Grande RS Brazil; ^8^ Laboratório de Ecologia e Conservação da Megafauna Marinha Universidade Federal do Rio Grande‐FURG Rio Grande RS Brazil

**Keywords:** Antarctic phytoplankton, light regimes, microcosms, non‐photochemical quenching, PHYTO‐PAM, pigment dynamics

## Abstract

Meltwater stratification during austral summer along the rapidly warming Western Antarctic Peninsula (WAP) exposes coastal phytoplankton to sudden light shifts. Such variability is thought to modulate phytoplankton dynamics, yet the photoacclimation capacity of Antarctic phytoplankton, especially cryptophytes, remains unclear. We grew a pennate diatom (*Fragilariopsis cylindrus*), a centric diatom (*Porosira glacialis*), and a cryptophyte (*Geminigera cryophila*) in microcosms subjected to low (30 μmol photons · m^−2^ · s^−1^), high (450 μmol photons · m^−2^ · s^−1^), and very low (6 μmol photons · m^−2^ · s^−1^) light over 24 days at 3°C, while monitoring cell growth, pigments, photo system II (PSII) quantum yield, and non‐photochemical quenching (NPQ). All taxa maintained positive growth (0.12–0.25 · d^−1^) and rapidly adjusted pigment ratios after light shifts, demonstrating efficient photoacclimation. The small pennate diatom reached 10‐fold higher cell densities than the centric diatom, although the latter accumulated more chlorophyll per cell and consumed twice as much inorganic nitrogen. *Geminigera cryophila* exhibited the most flexible short‐term NPQ response, dissipating over 60% of excess energy during high light pulses, yet this flexibility did not translate into any apparent advantage in growth over both diatoms during the period of the experiment. Field data showed cryptophyte dominance in shallower, more stratified waters, whereas diatoms prevailed in more mixed waters. Our results show that all three species can acclimate to Antarctica's complex light regime over days to weeks, yet cryptophytes may have a competitive advantage under sustained high light at shorter timescales. As warming and glacial melting continue, potentially favoring smaller cells over large bloom‐forming diatoms, it is crucial to understand how it will impact carbon export and trophic transfer.

**Abbreviations**
Allo: alloxanthin
Chl *a*
: chlorophyll *a*
Dd: diadinoxanthin
DIN: dissolved inorganic nitrogen
Dt: diatoxanthin
*E*k: minimum saturation irradiance
ETRmax: maximum electron transport rate
*F*
: fluorescence yield before saturation pulse
*F*m: maximum fluorescence yield of dark‐adapted sample
*F*m′: maximum fluorescence yield of light‐adapted sample
FURG: Federal University of Rio Grande
*F*_v_/*F*_m_
: maximum quantum yield of PSII
HL: high light
HPLC: high performance liquid chromatography
LL: low light
LSRE: light stress induction–recovery experiment
MLD: mixed‐layer depth
NPQ: nonphotochemical quenching
PAR: photosynthetically active radiation
PPC: photoprotective carotenoids
PSC: Photosynthetic carotenoids
PSII: photosystem II
rETR: relative electron transport rate
rETRmax: maximum relative electron transport rate
RLC: rapid light curve
ROS: reactive oxygen species
VLL: very low light
WAP: Western Antarctic Peninsula
Y(NO): Quantum yield of nonregulated non‐photochemical energy dissipation
Y(NPQ): Quantum yield of regulated non‐photochemical energy dissipation
Y(PSII): Quantum yield of photochemical energy conversion in PSII

## INTRODUCTION

The Western Antarctic Peninsula (WAP) is one of the fastest warming regions on the planet, making it a focal point for studying the impact of climate change on Antarctic marine ecosystems (Meredith & King, 2005; Siegert et al., 2019; Turner et al., 2005). Typically, the WAP experiences extreme seasonal cycles, with harsh winters characterized by limited sunlight and widespread sea ice formation, followed by summers with daylight extending beyond 20 h during which sea ice, ice shelves, and glaciers melt (Campbell & Aarup, 1989). During the austral summer, light availability in the upper water column can vary significantly due to rapid changes in cloud cover, snow, sea ice, icebergs, and meltwater‐induced stratification (Arrigo et al., 2017), particularly in the coastal areas where these factors interact.

Changes in light availability play a crucial role in modulating Antarctic phytoplankton dynamics. In early spring, following the darkness of the austral winter, light becomes the primary driver of early phytoplankton growth (Arrigo et al., 2017; Joy‐Warren et al., 2019). As summer approaches, the mixed‐layer depth gradually becomes shallower leading to the increase of light availability in the upper water column, promoting large biomass blooms (Arrigo et al., 2017; Costa et al., 2020; Ferreira et al., 2020). However, at the peak of summer, incident light may become excessive for phytoplankton, especially in meltwater‐influenced areas, where low salinity lenses can entrap cells in shallow upper layers (Alderkamp et al., 2011; Mendes et al., 2023; Petrou et al., 2016). The seasonal succession of phytoplankton communities in the WAP reflects these changes. In general, *Phaeocystis antarctica* (Haptophyta) and both centric and pennate diatoms frequently occur in early spring; late spring and early summer are marked by high biomasses of centric diatoms; cryptophytes increase during the summer peak; and autumn is marked by an increase in pennate diatoms and flagellates, including dinoflagellates (Costa et al., 2023; Ferreira et al., 2020; Joy‐Warren et al., 2019; Nardelli et al., 2023; Prézelin et al., 2004).

Several recent studies reported on the occurrence of phytoplankton compositional shifts during the summer peak (January–February), foremost an increasing predominance of cryptophytes over diatoms in coastal waters associated with well‐lit and shallow mixed layers influenced by meltwater (Mendes et al., 2013, 2018, 2023; Moline et al., 2004). Although the reasons for the seeming competitive advantage of cryptophytes are not yet fully understood, a few recent studies suggested that the advantage could reflect a greater capacity to endure high ambient light levels in a potentially more flexible and efficient way compared to diatoms (Mendes et al., 2018, 2023; Trimborn et al., 2019).

Diatoms and cryptophytes rely on distinct pigment‐based photoprotective strategies to cope with rapid changes in irradiance. In diatoms, excess light energy is primarily dissipated through the diadinoxanthin–diatoxanthin (Dd–Dt) xanthophyll cycle, which involves the reversible conversion of diadinoxanthin to diatoxanthin under high light (HL) and its reconversion under low light (LL). This cycle plays a central role in nonphotochemical quenching (NPQ) and photoprotection of the photosynthetic apparatus (Falkowski & Raven, 2013; Goss & Jakob, 2010). Cryptophytes, however, lack a xanthophyll cycle and instead rely on alternative mechanisms. In cryptophytes, alloxanthin, a major accessory carotenoid, has been shown to play a dual role in light harvesting under low irradiance and photoprotection under HL, potentially contributing to NPQ and excess energy dissipation (Henriksen et al., 2002; Laviale & Neveux, 2011; Mendes et al., 2023). Differences in the regulation of these pigment systems are expected to underpin contrasting photoacclimation strategies between diatoms and cryptophytes under fluctuating light regimes.

Diatoms are the primary prey of the Antarctic krill (*Euphausia superba*; Haberman et al., 2003; Pauli et al., 2021), which act as a critical link between phytoplankton and higher trophic levels in the Antarctic food web. Therefore, the shift from diatoms to cryptophyte‐dominated communities could potentially have significant implications for the Antarctic marine ecosystems (Ferreira et al., 2020; Moline et al., 2004; Schofield et al., 2017).

Previous studies have already investigated the response of Antarctic cryptophytes and diatoms to different light intensities (e.g., Camoying & Trimborn, 2023; Heiden et al., 2016; Mendes et al., 2023; Trimborn et al., 2019). Although results varied among different species used and depending on the tested light conditions, the consensus was that ambient light intensity is an important factor modulating Antarctic phytoplankton growth and carbon uptake. Species‐specific responses have also been observed, with centric diatoms being less vulnerable to prolonged HL periods than pennate diatoms, whereas cryptophyte responses to HL can be highly dependent on the ambient temperature (favoring high temperatures, >2°C; Camoying & Trimborn, 2023; Heiden et al., 2016; Mendes et al., 2023; Trimborn et al., 2019). Nonetheless, most studies have focused on the phytoplankton response under a single continuous period of a given light intensity, which is insufficient to resolve the full range of ecological responses by phytoplankton to light variations caused by cloud cover, precipitation, and melt‐enhanced stratification that the cells endure in the shallow mixed layers of the Antarctic summer.

To address this gap, we conducted a 24‐day laboratory microcosm experiment that subjected three Antarctic taxa—a pennate diatom, a centric diatom, and a cryptophyte—to successive phases of low (30 μmol photons · m^−2^ · s^−1^), high (450 μmol photons · m^−2^ · s^−1^), and very low (6 μmol photons · m^−2^ · s^−1^) irradiance at 3°C, aimed at simulating their complex ambient light environment. After each light shift, we assessed the growth rates, pigmentary changes, and photophysiological responses of each species. Our main goal was to understand how Antarctic cryptophytes and diatoms cope with rapid light fluctuations and whether cryptophytes gain a competitive edge under sustained HL that could be driving their emergence along coastal WAP.

## MATERIALS AND METHODS

### Phytoplankton species

Three Antarctic phytoplankton species were obtained from the Phytoplankton Laboratory Microalgae Culture Collection at the Federal University of Rio Grande (FURG): a centric diatom (*Porosira glacialis*), a pennate diatom (*Fragilariopsis cylindrus*), and a cryptophyte (*Geminigera cryophila*). All species were isolated during austral summer cruises carried out in the Bransfield Strait (ca. 63° S, 59° W) and maintained ever since in L1‐enriched medium (Guillard & Hargraves, 1993) prepared with filtered seawater from the same region, at 3°C, a salinity of 35, and under a 14:10 h light:dark cycle (30–40 μmol photons · m^−2^ · s^−1^).

*Porosira glacialis* is among the most abundant centric diatoms along the WAP, especially in coastal waters adjacent to sea ice (Hasle, 1976; Olguín & Alder, 2011; Pike et al., 2009). The strain used here averaged 27 μm in apical and 19 μm in trans‐apical axes, with a mean biovolume of 10,900 μm^3^. *Fragilariopsis cylindrus*, a pennate diatom associated with pack ice and ice‐edge blooms (Cefarelli et al., 2010; Hasle & Syvertsen, 1997), was far smaller, measuring 5.2 × 2.6 μm (55 μm^3^). Finally, *Geminigera cryophila* is the main cryptophyte species in the WAP, occurring in warm, melt‐stratified coastal waters and often outcompeting diatoms under those conditions (Mendes et al., 2018; Trefault et al., 2021). The strain used in these experiments exhibited an average size of 10 μm in length and 5 μm in width (biovolume = 262 μm^3^).

### Experimental setup

Each species was pre‐acclimated for 2 weeks in 200‐mL Erlenmeyer flasks at 3°C, a salinity of 35, and a 14:10 h light:dark cycle under LL (~30 μmol photons · m^−2^ · s^−1^), reflecting the typical low‐light environment of the WAP (Dierssen et al., 2000; Smith et al., 1996). After reaching the exponential growth phase, ~5000 cells · mL^−1^ of each species were inoculated into 200‐mL Erlenmeyers, hereafter referred to as microcosms, containing filtered Bransfield Strait seawater enriched with L1 culture medium (Guillard & Hargraves, 1993). Bulk medium was prepared in a single 2.5‐L carboy and then divided into 200‐mL microcosms to ensure identical starting nutrient concentrations (883 μM NO_3_^−^, 36 μM PO_4_^3−^, 170 μM Si[OH]_4_, plus other essential micronutrients included in the L1 medium). The experiment was run in triplicates.

**FIGURE 1:**
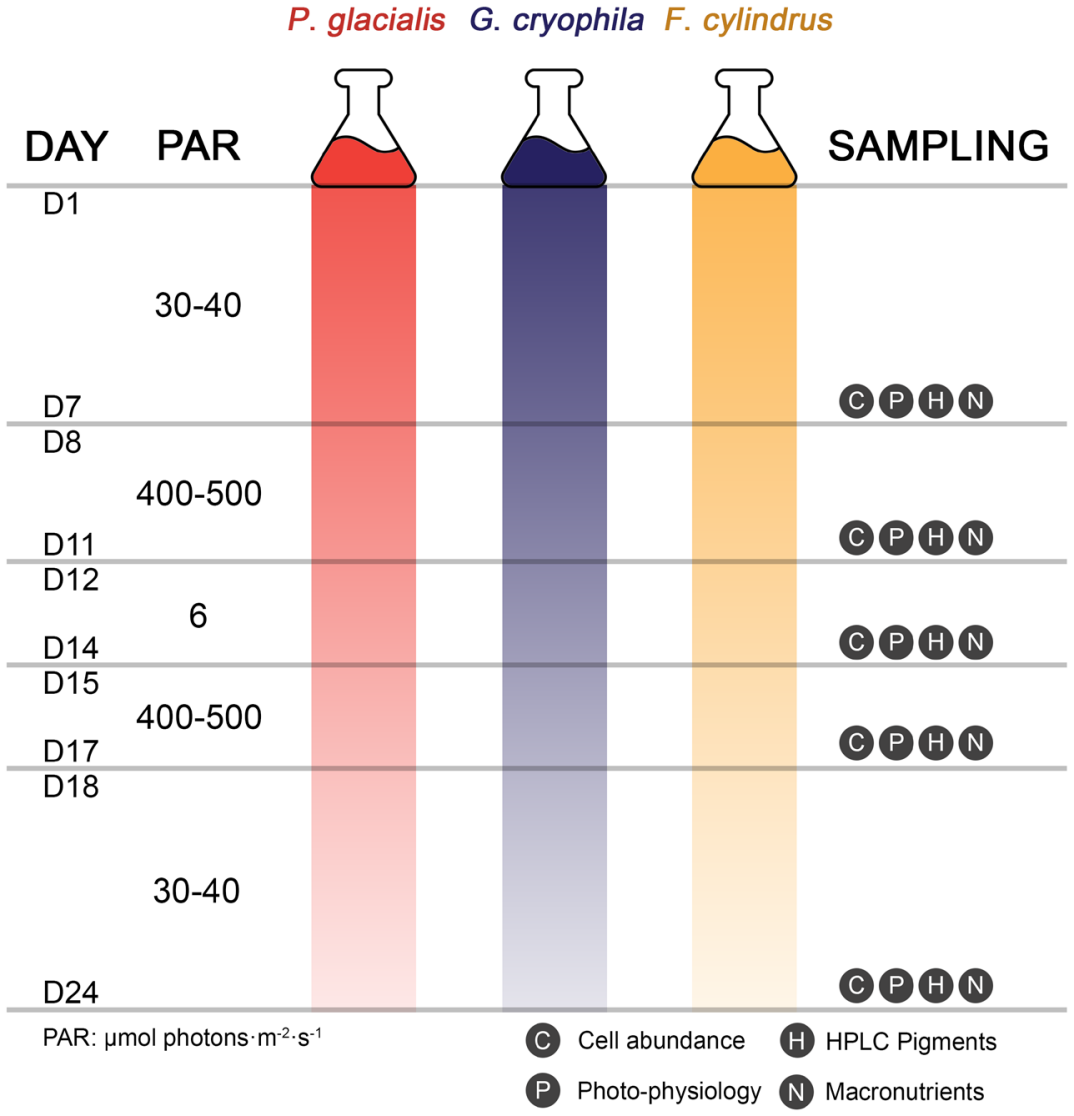
Depiction of the experimental setup since Day 1 (D1) to Day 24 (D24). For each stage, the light intensities that the species were subjected to are shown (very low = 6 μmol photons · m^−2^ · s^−1^, low = 30–40 μmol photons · m^−2^ · s^−1^, high = 400–500 μmol photons · m^−2^ · s^−1^). On the last day of each phase, sampling was conducted for the following analyses: Cell abundance estimates via light microscopy (C), photophysiological response assessment using PHYTO‐PAM (P), determination of phytoplankton pigments using high performance liquid chromatography (H), and quantification of macronutrients (N). Each species' treatment was run in triplicates. Light intensity variation throughout the experiment is further illustrated in Figure S1.

In order to simulate the rapid light swings often observed in coastal WAP, the microcosms were subjected to five sequential irradiance phases: (i) Days 1–7: 7 days under LL (~30–40 μmol photons · m^−2^ · s^−1^), (ii) Days 8–11: 4 days under HL (~400–500 μmol photons · m^−2^ · s^−1^), (iii) Days 12–14: 3 days under very low light (VLL, ~6 μmol photons · m^−2^ · s^−1^), (iv) Days 15–17: 3 days under HL, and finally, (v) Days 18–24: 7 days under LL (Figure 1). These light levels were in accordance with the wide range of in situ photosynthetically active radiation (PAR) measured in stratified summer WAP surface layers (from 0 to 80 μmol photons · m^−2^ · s^−1^ to >500 μmol photons · m^−2^ · s^−1^; Joy‐Warren et al., 2019; Russo et al., 2018; Venables et al., 2013), marked by frequent days with high cloud coverage and sea ice occurrence (leading to low ambient light) and occasional days with clear sky that increase PAR substantially. White light fluorescent lamps (Osram®) were used to provide the various irradiance levels tested in this experiment. To achieve the lower irradiance levels, the Erlenmeyer flasks were covered with mesh screens to attenuate the incident light without changing the light spectrum. A 14:10 h (on:off) light cycle was maintained throughout the experiment (Figure S1). The irradiance at the center of the microcosm was measured daily with a Spherical Micro Quantum Sensor (Walz GmbH®).

Sampling was conducted every 2 days, to monitor cell abundance and photosynthetic efficiency (5 mL samples), and whenever the microcosms transitioned to a new irradiance level, to measure cell abundance, photophysiology, phytoplankton pigments, and macronutrients (28 mL samples; days 7, 11, 14, 17, and 24). Sampling was always conducted during the morning. The microcosms were manually agitated prior to sampling and two times per day. The extracted volume was not replaced, leaving ~63 mL at the end of the experiment. Temperature, salinity, and photoperiod remained constant throughout the experiment.

### Laboratory analysis

#### Cell abundance and growth

Three‐milliliter samples were taken and preserved with 1% neutral Lugol solution. These samples were then used for cell counting under an inverted light microscope (OLYMPUS IX51) using Sedgewick Rafter counting chambers. A minimum of 10 grid cells of the counting chamber were examined. When high variability in cell counts was observed, at least 15 grid cells were counted to improve precision. Growth rates (μ) were calculated using Equation (1):

(1) $$\mu =\ln\left(\frac{N_{t}}{N_{0}}\right)\times \frac{1}{t}$$

where ln is the natural logarithm, *t* is the time elapsed (in days), and *N*_*t*_ and *N*_0_ are the final and initial cell abundances, respectively.

#### Photophysiology

Maximum photosystem II (PSII) quantum yields (*F*_v_/*F*_m_), a proxy of photosynthetic efficiency, were measured every other day with a pulse‐amplitude modulated Phyto‐PAM fluorometer (Walz GmbH). Prior to the measurement, 2‐mL samples were kept in the dark for 1 h to ensure that the PSII reaction centers were fully open (Schreiber et al., 1995). On days 7, 11, 14, 17, and 24, we also ran rapid light curves (RLCs) and light‐stress induction–recovery experiments (LSREs) following Mendes et al. (2023).

The RLCs consisted of exposing a dark‐adapted 2‐mL sample to 21 successive, incremental light 1‐min steps ranging from 0 to 650 μmol photons · m^−2^ · s^−1^. At the end of each step, the steady‐state fluorescence (*F*′) and the saturating‐pulse‐induced maximum fluorescence (*F*m′) were measured. The effective PSII quantum yield was calculated as Y(PSII) = (*F*m′ – *F*′)/*F*m′. The fraction of energy dissipated as heat via regulated non‐photochemical quenching, Y(NPQ), was calculated as Y(NPQ) = *F*/*F*m′ – *F*/*F*m. The fraction of energy dissipated as heat via nonregulated mechanisms, Y(NO), was calculated as Y(NO) = *F*/*F*m. Note that the Y(PSII) + Y(NPQ) + Y(NO) = 1, since each corresponds to a fraction of the total absorbed excitation energy in PSII that can either be used in photosynthesis, Y(PSII), or lost, Y(NPQ) and Y(NO). The relative electron transport rate (rETR) was calculated as rETR = Y(PSII) · *E*, where *E* is the PAR at the light step. The rETR and *E* were fit to the empirical model described by Platt et al. (1980), which allowed for deriving the following RLC parameters: (i) the initial slope of the RLC at limiting irradiance (α), indicating the light utilization efficiency; (ii) the maximum relative electron transport rate during the light curve (rETR_max_); and (iii) β, a photoinhibition parameter that characterizes the downturn of the RLC after reaching rETRmax. Using α and rETRmax, the light saturation coefficient (*E*k; μmol photons · m^−2^ · s^−1^) was calculated as rETRmax/α. A full list of the equations is included in Table S1.

The LSREs were always run after the RLCs using an independent dark‐adapted 2‐mL sample as follows: (i) one measurement in complete darkness, (ii) four measurements during 3 min under LL (14 μmol photons · m^−2^ · s^−1^) to allow for the dissipation of NPQ that might have accumulated in darkness (Jesus et al., 2006), (iii) nine measurements during 9 min under HL (650 μmol photons · m^−2^ · s^−1^), (iv) four measurements during 2 min under LL, and finally, (v) 21 measurements during 15 min in complete darkness. Each LSRE session lasted ~29 min. Calculations for Y(PSII), Y(NPQ), and Y(NO) were done for each measurement or step of the LSRE, following the same methodology as for the RLCs.

All measurements were conducted with the Phyto‐PAM fluorometer using 2‐mL samples in a stirred chamber at a constant temperature of 3°C. All calculations required were performed in R 4.3.3 (R Core Team, 2024).

#### High performance liquid chromatography phytoplankton pigment analysis

Twenty‐milliliter samples were filtered using Whatman® GF/F filters (25‐mm diameter; 0.7‐μm pore size) and immediately frozen at −80°C. Prior to analysis in the laboratory, 3 mL of 95% cold‐buffered methanol containing 0.05 mg · L^−1^ trans‐β‐apo‐8′‐carotenal (Fluka) as an internal standard were added to the filter. Samples were then sonicated (5 min in an ice‐water bath), placed at −20°C for 1 h, and centrifuged (5 min at 3°C; 1100 × *g*). Supernatants were filtered through Fluoropore PTFE membrane filters (0.2‐μm pore size) to remove any residual filter and cell debris. Prior to injection, 1000 μL of the sample were mixed with 400 μL of Milli‐Q water in 2‐mL vials.

Pigment identification and quantification was performed using a Shimadzu high performance liquid chromatography (HPLC) system equipped with a solvent distributor (LC‐20AD), a control system (CBM‐20M), a photodiode detector (SPDM‐20A), and a fluorescence detector (RF‐10AXL). A monomeric C8 column (SunFire; 15‐cm length; 4.6‐mm diameter; 3.5‐μm particle size) was used for pigment separation, following protocols described by Zapata et al. (2000) and Mendes et al. (2007). Pigment concentrations were determined from signals detected by the photodiode array detector, using commercial standards from DHI Denmark. Peak integration was performed using LC‐Solution software, with manual verification and correction as needed. To account for losses and volume changes, pigment concentrations were normalized to the internal standard. Quality assurance procedures, using quantification limit (LOQ) and detection limit (LOD), followed the methodology outlined by Mendes et al. (2007).

To assess pigment photoacclimation, carotenoids were separated into photosynthetic carotenoids (PSCs) and photoprotective carotenoids (PPCs) and used to calculate PPC:PSC ratios. The PSCs included fucoxanthin, and PPCs comprised alloxanthin, crocoxanthin, diadinoxanthin, diatoxanthin, β‐carotene, and α‐carotene (Araujo et al., 2017). The ratio between PPC and chlorophyll *a* (PPC:Chl *a*) was also calculated. For both PPC:PSC and PPC:Chl *a*, higher values indicate photoacclimation.

Moreover, the diatoxanthin:diadinoxanthin (Dt:Dd) ratio was calculated for *Porosira glacialis* and *Fragilariopsis cylindrus*, as an indicator of xanthophyll‐cycle activity in diatoms. For the cryptophyte *Geminigera cryophila*, which lacks a xanthophyll cycle, the alloxanthin:chlorophyll *a* (Allo:Chl *a*) ratio was calculated to evaluate changes in auxiliary pigment allocation.

#### Dissolved inorganic macronutrients analysis

Dissolved inorganic nitrate (NO_3_^−^), nitrite (NO_2_^−^), phosphate (PO_4_^3−^), and silicic acid [Si(OH)_4_] concentrations were quantified using a SEAL Analytical Autoanalyzer AA3 HR (software version 7.10; Grasshoff et al., 2009). To this end, 20‐mL samples were filtered through cellulose acetate membrane filters and then frozen at −20°C. Measurements of Si(OH)_4_ were corrected for sea salt interference, whereas PO_4_^3−^ was quantified through its reaction with ammonium molybdate, with absorption readings taken at 885 nm (Grasshoff et al., 2009). The detection limits were 0.006 μmol · L^−1^ for NO_3_^−^ and NO_2_^−^ and 0.016 μmol · L^−1^ for PO_4_^3−^ and Si(OH)_4_. Dissolved inorganic nitrogen (DIN) was calculated as NO_3_^−^ + NO_2_^−^.

### In situ analysis of the environmental niches of diatoms and cryptophytes

To complement the laboratory experiments and further understand the environmental conditions associated with centric diatoms, pennate diatoms, and cryptophytes, we analyzed pigment‐based chemotaxonomy, cell abundance, and environmental data from 2008 to 2019 WAP cruises (*N* = 175). CHEMTAX (Mackey et al., 1996) was used to identify in situ stations with high abundance (>50% of the total biomass) of either diatoms or cryptophytes, which was possible since diatoms and cryptophytes exhibit distinct suites of phytoplankton pigments. For diatom‐dominated stations, cell abundance was used to differentiate between stations dominated by either centric or pennate diatoms. Finally, the environmental conditions for each subset (centric diatom‐dominated, pennate diatom‐dominated, and cryptophyte‐dominated) were compared.

The in situ data were obtained over the course of 13 austral summer expeditions to the WAP aboard the Brazilian vessels NP Almirante Maximiano and NP Ary Rongel (2008–2019), led by the Brazilian High‐Latitude Oceanography Group (GOAL‐FURG). A Sea–Bird® CTD (conductivity–temperature–depth instrument) and a Carrousel 911+ system equipped with twenty‐four 12‐L Niskin bottles was used to measure in situ temperature (°C) and practical salinity. The seawater potential density (kg · m^−3^) referenced to 0 dbar was determined based on seawater conservative temperature, absolute salinity, and pressure data using the Thermodynamic Equation of Seawater – 2010 (TEOS‐10). Seawater potential density was then used to estimate the mixed layer depth (MLD; m) and the water column stability (10^−6^ m), two key indicators of the water column physical structure. The MLD was calculated according to the criteria established by de Boyer Montégut et al. (2004), whereas the water column stability was estimated through the Brünt–Väisälä frequency (N^2^), following previous works for the same region by Costa et al. (2020, 2021).

For cell counts and taxonomic identification via light microscopy, 250 mL of near‐surface (5 m) seawater were sampled from each station and preserved with 1% neutral Lugol solution. Cell counting followed the same principles described above. For HPLC pigment determination, near‐surface seawater samples (ranging from 0.5 to 2.5 L) were filtered under low vacuum, stored at −80°C, and then analyzed following the same techniques described above. Using Chl *a* degradation products—that is, chlorophyllide *a* (Chlide *a*), pheophytin *a* (Phytin *a*), and pheophorbide *a* (Phide *a*)—quantified through HPLC, it was possible to calculate two proxies that are helpful in understanding the status of the phytoplankton community at sampling. In this sense, the formula (Phytin *a* + Phide *a*)/TChl *a* × 100 was used as a grazing index (%), which is an indication of the grazing pressure the community was under prior to sampling. The senescence of phytoplankton cells, that is, their natural degradation, was estimated using a senescence index based on Chlide *a*: Chlide *a*/TChl *a* × 100 (%). Both indices have been used for the WAP and have been shown to be ecologically relevant (Costa et al., 2020, 2021).

### Statistical analysis

Pigment profiles of each species throughout the experiment were clustered with agglomerative hierarchical clustering and Ward's method (Ward Jr, 1963) using the scikit‐learn module (Pedregosa et al., 2011). Prior to the analysis, pigment data was standardized (−1 to 1). Mann–Whitney *U* tests were also used throughout the experiment to assess the existence of statistically significant differences between species' responses. Kruskal–Wallis tests were used to identify differences between the environmental properties of the in situ stations dominated by centric diatoms, pennate diatoms, and cryptophytes, along with pairwise multiple comparison.

## RESULTS

### Growth and nutrient consumption

**FIGURE 2:**
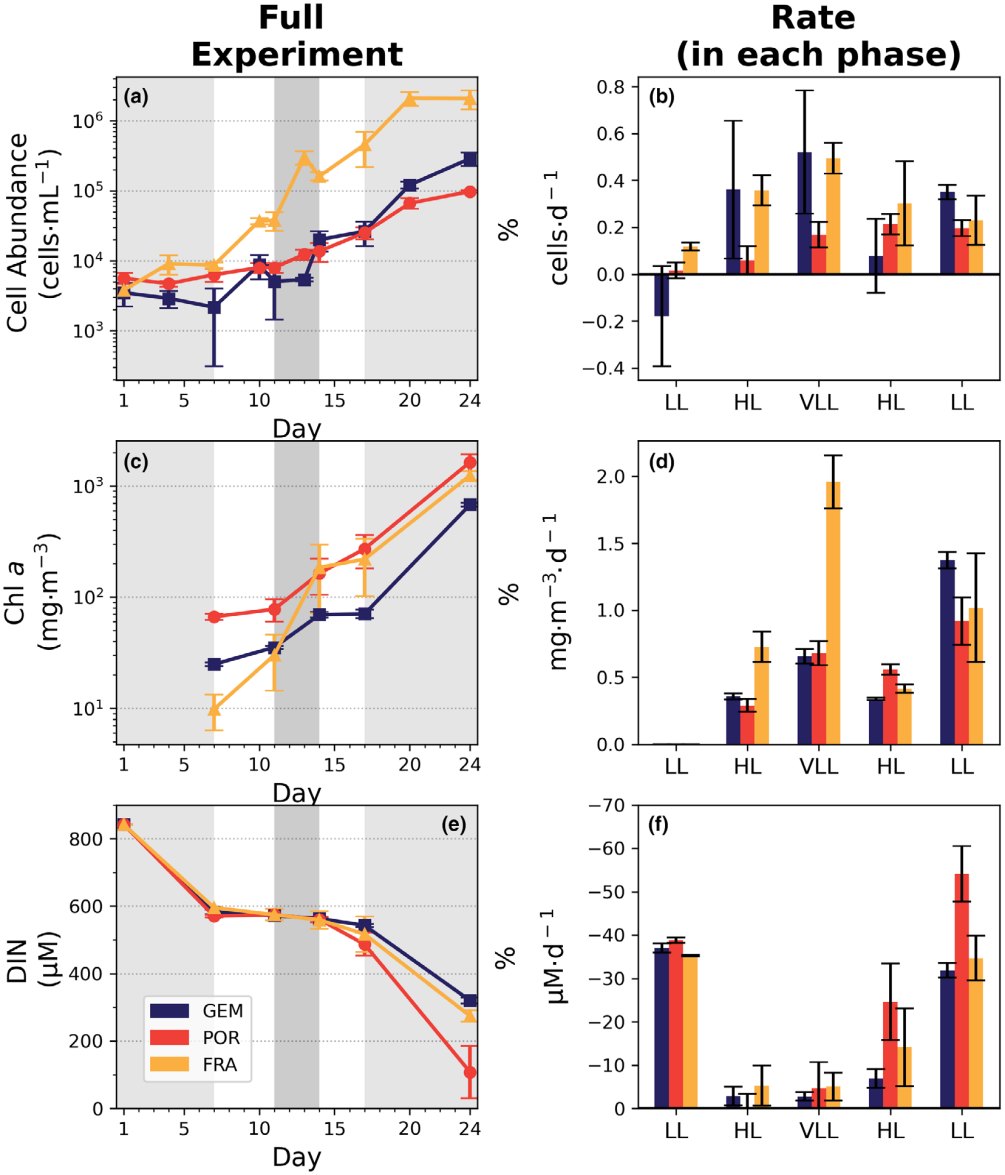
Cell abundance (cells · mL^−1^), chlorophyll *a* concentration (Chl *a*; mg · m^−3^; only measured from Day 7 onward), and dissolved inorganic nitrogen (DIN; μM) throughout the treatment. For each parameter, the absolute measured values (a, c, and e panels) and the daily rate of growth, increase or consumption since the previous light phase (d^−1^; b, d, and f panels) are presented. Each value represents the mean ± standard deviation of three replicates (*n* = 3). Statistically significant differences among the species were tested using Kruskal–Wallis and Mann–Whitney tests. Note that the growth rates calculated for cell abundance and Chl *a* have been log‐normalized. Shaded areas in panels a, d, and g represent the periods under the low (30–40 μmol photons · m^−2^ · s^−1^) and very low (6 μmol photons · m^−2^ · s^−1^) light stages. FRA, *Fragilariopsis cylindrus*; GEM, *Geminigera cryophila*; POR, *Porosira glacialis*.

All species exhibited substantial growth and peaked in day 24, although some interspecific differences were observed throughout (Table 1; Figure 2). *Fragilariopsis cylindrus* reached a maximum cell abundance of 2.1 ± 0.6 × 10^6^ cells · mL^−1^, over 10‐fold the peak abundance observed for both *Geminigera cryophila* (2.9 ± 0.6 × 10^5^ cells · mL^−1^) and *Porosira glacialis* (9.7 ± 0.4 × 10^4^ cells · mL^−1^; Figure 2a). Growth rates (μ; Figure 2b) for the full period ranged between 0.12 and 0.26 · d^−1^. Nevertheless, phase‐specific growth rates were highest for *F. cylindrus* and *G. cryophila* under VLL (0.5 · d^−1^ and 0.52 · d^−1^), whereas the growth rate of *P. glacialis* peaked during the second high light pulse (0.21 · d^−1^).

**TABLE 1:** Cell abundance (Cells; cells · mL^−1^), chlorophyll *a* (Chl *a*; mg · m^−3^), *F*_v_/*F*_m_, dissolved inorganic nitrogen (DIN; μM), phosphate (μM), and silicic acid (μM) measure during the treatments with each species.

Species	Parameters	D1 (LL)	D4 (LL)	D7 (LL)	D10 (HL)	D11 (HL)	D13 (VLL)	D14 (VLL)	D17 (HL)	D20 (LL)	D24 (LL)
**(a)**											
*Geminigera cryophila*	Cells	3500 ± 1296	2900 ± 804*	2167 ± 1855*	8767 ± 3289	5067 ± 3638*	5367 ± 309*	20,033 ± 6402*	26,233 ± 10,031*	120,833 ± 14432*	287,067 ± 63,936*
	Chl *a*			24.90 ± 0.98*		35.32 ± 1.12*		69.48 ± 4.23*	70.66 ± 6.13*		676.83 ± 28.90*
	*F*_v_/*F*_m_	0.58 ± 0.01	0.64*	0.67 ± 0.01*	0.39 ± 0.04	0.39 ± 0.03*	0.61 ± 0.01*	0.66 ± 0.02	0.29 ± 0.03*	0.62	0.7 ± 0.01*
	Nitrate			583 ± 3.34*		571.56 ± 1.11		563.35 ± 2.02	542.49 ± 4.38*		319.91 ± 8.64*
	Nitrite			0		0		0*	0.03 ± 0.02		0
	Phosphate			27.1 ± 0.89		24.66 ± 0.78		22.56 ± 1.26	18.85 ± 0.57		2.05 ± 0.78
	Silicic acid			177.78 ± 4.68*		177.78 ± 7.22*		184.01 ± 1.24	189.21 ± 10.94*		180.46 ± 10.36*
	N:P			21.53 ± 0.67		30.16 ± 10.25		47.09 ± 1.24*	28.82 ± 1.03		172 ± 55.12*
	N:Si			3.28 ± 0.67		3.05 ± 10.25		3.06 ± 1.24	2.88 ± 1.03		1.78 ± 55.12
**(b)**											
*Porosira glacialis*	Cells	5633 ± 984	4700 ± 424*	6333 ± 1360*	7967 ± 1297	7967 ± 1320*	12,333 ± 2042*	13,667 ± 4066*	25,133 ± 4940*	66,133 ± 11,372*	97,400 ± 4306*
	Chl *a*			66.59 ± 4.17*		77.78 ± 17.75*		163.50 ± 58.24*	271.57 ± 89.64*		1635.93 ± 277.12*
	F_v_/F_m_	0.32 ± 0.02	0.44*	0.5*	0.41 ± 0.01	0.35 ± 0.02*	0.65 ± 0.01*	0.68 ± 0.01	0.31 ± 0.02*	0.65*	0.65*
	Nitrate			570.32 ± 4.17*		570.324 ± 4.17		560.35 ± 8.16	486.58 ± 34.09*		107.88 ± 77.31*
	Nitrite			0.01 ± 0.01		0.1 ± 0.03		0.01 ± 0.01*	0.01 ± 0		0
	Phosphate			30.45 ± 0.23		28.01 ± 0.77		24.26 ± 1.74	13.84 ± 4.46		0.55 ± 0.45
	Silicic acid			220.18 ± 10.30*		215.361 ± 28.97*		210.98 ± 13.99	169.13 ± 22.67*		6.73 ± 2.49*
	N:P			25.87 ± 8.13		21.41 ± 4.11		36.22 ± 10.54*	46.41 ± 21.27		374.31 ± 387.44*
	N:Si			2.6 ± 0.11		2.71 ± 0.32		2.66 ± 0.14	2.9 ± 0.18		13.98 ± 5.62
**(c)**											
*Fragilariopsis cylindrus*	Cells	3800 ± 455	9100 ± 2782*	8633 ± 785	36,967 ± 3952	37,867 ± 11,172*	300,266 ± 66,956*	160,967 ± 21,773*	454,467 ± 237,550*	2,089,800 ± 455,757*	2,073,067 ± 630,243*
	Chl *a*			9.83 ± 3.50		30.11 ± 15.72*		184.62 ± 113.30*	218.87 ± 116.36*		1243.30 ± 117.65*
	F_v_/F_m_	0.34 ± 0.03	0.41 ± 0.03*	0.59 ± 0.04	0.19 ± 0.03	0.20 ± 0.04*	0.38 ± 0.08*	0.71 ± 0.06	0.22 ± 0.05*	0.57 ± 0.11	0.57 ± 0.02*
	Nitrate			595.22 ± 1.5		574.19 ± 17.24		558.91 ± 26.16	516.56 ± 53.01*		273.78 ± 17.37*
	Nitrite			0.03 ± 0.01		0.09 ± 0.03		0.21 ± 0.05*	0.31 ± 0.1		0.44 ± 0.07
	Phosphate			30.12 ± 3.53		27.86 ± 5.42		22.75 ± 7.54	14.27 ± 9.43		0.47 ± 0.16
	Silicic acid			217.57 ± 18.92*		215.36 ± 28.97*		168.99 ± 46.37	115.25 ± 66.68*		2.47 ± 0.13*
	N:P			20.06 ± 2.58		21.41 ± 4.11		28.09 ± 10.63*	167.49 ± 199.26		636.61 ± 188.49*
	N:Si			2.76 ± 0.26		2.71 ± 0.32		3.59 ± 1.03	9.28 ± 8.29		111.49 ± 12.57

*Note*: The table is subdivided into three subtables, each presenting data for a single species: *Geminigera cryophila* (a), *Porosira glacialis* (b), and *Fragilariopsis cylindrus* (c). The initial estimated macronutrient concentrations (Day 0) were: nitrate = 883 μM, nitrite = 0 μM, phosphate = 36 μM, and silicic acid = 170 μM. Standard deviations <0.01 units are not shown.

* Statistically significant differences among species on the same sampling day (Kruskal–Wallis test, *p* < 0.05).

Despite exhibiting significantly lower cell abundance by day 24, *Porosira glacialis* accumulated a higher amount of Chl *a* owing to its larger biovolume (Figure 2c,d; Figures S2 and S3): 1.6 ± 0.3 g · m^−3^, ~2.4× higher than *Geminigera cryophila* and ~1.31× higher than pennate diatom *Fragilariopsis cylindrus*. In terms of intracellular Chl *a*, this corresponded to 16.9 ± 3.4 pg Chl *a* · cell^−1^ for *P. glacialis*, 2.5 ± 0.5 pg Chl *a* · cell^−1^ for *G. cryophila*, and 0.6 ± 0.1 pg Chl *a* · cell^−1^ for *F. cylindrus* (Table 2). Both diatoms always reduced their cellular Chl *a* when light shifted from low to high (−26% on average). *Geminigera cryophila*, however, initially increased its cellular Chl *a* during the first LL‐to‐HL shift (+13%; Day 11), yet later decreased Chl *a* when moving from VLL to HL (−18%; Day 17).

**TABLE 2:** Pigmentary composition of *Geminigera cryophila*, *Porosira glacialis*, and *Fragilariopsis cylindrus* for each light phase of the treatment.

	LL (D7)	HL (D11)	VLL (D14)	HL (D17)	LL (D24)
*Geminigera cryophila*					
Chl *a*	24.26 ± 0.40	35.32 ± 1.12	69.48 ± 4.23	70.66 ± 6.13	676.83 ± 28.9
Chl *a* (pg per cell)	9.44 ± 4.26	10.64 ± 5.15	3.83 ± 1.23	3.13 ± 1.18	2.46 ± 0.48
Allo	8.08 ± 0.08	22.08 ± 0.71	26.15 ± 1.98	49.75 ± 2.41	204.49 ± 12.85
Allo (pg per cell)	3.14 ± 1.40	6.79 ± 4.50	1.44 ± 0.46	2.24 ± 0.94	0.74 ± 0.14
Chl *c*_1_	0	0	0	0	0
Chl *c*_1_ (pg per cell)	0	0	0	0	0
Chl *c*_2_	3.83 ± 0.09	5 ± 0.16	11.23 ± 0.7	12.29 ± 0.59	112.87 ± 4.77
Chl *c*_2_ (pg per cell)	1.47 ± 0.61	1.54 ± 0.78	0.62 ± 0.2	0.56 ± 0.23	0.41 ± 0.08
Croco	1.07 ± 0.08	1.38 ± 0.02	1.78 ± 0.09	2.6 ± 0.12	24.72 ± 1.65
Croco (pg per cell)	0.43 ± 0.21	0.42 ± 0.21	0.1 ± 0.04	0.12 ± 0.05	0.09 ± 0.02
Fuco	0	0	0	0	0
Fuco (pg per cell)	0	0	0	0	0
Diadino	0	0	0	0	0
Diadino (pg per cell)	0	0	0	0	0
Diato	0	0	0	0	0
Diato (pg per cell)	0	0	0	0	0
α‐car	1.36 ± 0.01	1.93 ± 0.05	3.18 ± 0.22	3.94 ± 0.3	35.76 ± 0.78
α‐car (pg per cell)	0.54 ± 0.23	0.59 ± 0.3	0.17 ± 0.05	0.18 ± 0.07	0.13 ± 0.03
β‐car	0	0	0	0	0
β‐car (pg per cell)	0	0	0	0	0
Allo:Chl *a*	0.33 ± 0	0.63 ± 0.04	0.38 ± 0.01	0.71 ± 0.03	0.30 ± 0.01
PPC:PSC					
PPC:Chl *a*	0.43	0.72 ± 0.04	0.45	0.80 ± 0.03	0.39 ± 0.01
*Porosira glacialis*					
Chl *a*	66.59 ± 4.17	77.78 ± 17.75	163.5 ± 58.24	271.57 ± 89.64	1635.93 ± 277.12
Chl *a* (pg per cell)	11.05 ± 2.66	9.71 ± 1.13	11.82 ± 0.98	10.5 ± 1.64	16.93 ± 3.39
Allo	0	0	0	0	0
Allo (pg per cell)	0	0	0	0	0
Chl *c*_1_	4.18 ± 0.23	3.77 ± 0.66	8.45 ± 2.67	11.17 ± 3.5	77.86 ± 10.43
Chl *c*_1_ (pg per cell)	0.69 ± 0.16	0.47 ± 0.03	0.62 ± 0.03	0.43 ± 0.06	0.81 ± 0.14
Chl *c*_2_	4.13 ± 0.39	4.09 ± 1.13	11.45 ± 4.29	16.1 ± 5.47	131.35 ± 24.85
Chl *c*_2_ (pg per cell)	0.68 ± 0.17	0.51 ± 0.08	0.82 ± 0.07	0.62 ± 0.1	1.36 ± 0.3
Croco	0	0	0	0	0
Croco (pg per cell)	0	0	0	0	0
Fuco	32.5 ± 1.82	30.9 ± 6.11	79.89 ± 27.67	114.78 ± 40.65	789.88 ± 124.14
Fuco (pg per cell)	5.4 ± 1.29	3.89 ± 0.43	5.78 ± 0.37	4.41 ± 0.81	8.17 ± 1.54
Diadino	8.17 ± 0.17	8.99 ± 1.81	13.28 ± 3.29	31.35 ± 9.8	157.08 ± 27.27
Diadino (pg per cell)	1.35 ± 0.29	1.13 ± 0.1	1 ± 0.13	1.23 ± 0.17	1.63 ± 0.33
Diato	0.59 ± 0.02	12.94 ± 3.42	1.02 ± 0.22	56.94 ± 21.59	6.97 ± 2.48
Diato (pg per cell)	0.1 ± 0.02	1.61 ± 0.23	0.08 ± 0.01	2.18 ± 0.48	0.08 ± 0.03
α‐car	0	0	0	0	0
α‐car (pg per cell)	0	0	0	0	0
β‐car	1.98 ± 0.15	5.15 ± 0.91	5.3 ± 1.39	13.02 ± 2.80	45.68 ± 8.4
β‐car (pg per cell)	0.33 ± 0.08	0.65 ± 0.05	0.38 ± 0.04	0.52 ± 0.07	0.47 ± 0.1
Diato:Diadino	0.07	1.42 ± 0.09	0.08	1.8 ± 0.47	0.04 ± 0.01
PPC:PSC	0.33 ± 0.01	0.87 ± 0.04	0.25 ± 0.03	0.9 ± 0.05	0.26 ± 0.01
PPC:Chl *a*	0.16 ± 0.01	0.35	0.12 ± 0.01	0.38 ± 0.01	0.13
*Fragilariopsis cylindrus*					
Chl*a*	9.83 ± 3.5	30.11 ± 15.72	184.62 ± 113.3	218.87 ± 116.36	1243.3 ± 117.65
Chl *a* (pg per cell)	1.12 ± 0.31	0.83 ± 0.38	1.11 ± 0.59	0.5 ± 0.12	0.64 ± 0.13
Allo	0	0	0	0	0
Allo (pg per cell)	0	0	0	0	0
Chl *c*_1_	1.03 ± 0.34	1.7 ± 0.92	19.62 ± 11.68	18.27 ± 9.55	130.44 ± 11.64
Chl *c*_1_ (pg per cell)	0.12 ± 0.04	0.05 ± 0.02	0.12 ± 0.06	0.04 ± 0.01	0.07 ± 0.01
Chl *c*_2_	0.63 ± 0.24	1.64 ± 0.93	12.61 ± 7.8	13.59 ± 7.54	85.66 ± 11.52
Chl *c*_2_ (pg per cell)	0.07 ± 0.02	0.04 ± 0.02	0.08 ± 0.04	0.03 ± 0.01	0.04 ± 0.01
Croco	0	0	0	0	0
Croco (pg per cell)	0	0	0	0	0
Fuco	6.23 ± 2.16	13.90 ± 7.42	102.95 ± 61.64	107.31 ± 57.8	677.28 ± 65.25
Fuco (pg per cell)	0.71 ± 0.19	0.38 ± 0.18	0.62 ± 0.32	0.25 ± 0.06	0.35 ± 0.08
Diadino	0.62 ± 0.18	2.34 ± 1.64	8.74 ± 6.07	10.03 ± 4.51	73.83 ± 9.82
Diadino (pg per cell)	0.07 ± 0.02	0.06 ± 0.04	0.05 ± 0.03	0.02 ± 0.01	0.04 ± 0.01
Diato	0	6.78 ± 3.5	0.69 ± 0.46	56.38 ± 32.04	41.26 ± 44.84
Diato (pg per cell)	0	0.19 ± 0.08	0	0.13 ± 0.03	0.02 ± 0.02
α‐car	0	0	0	0	0
α‐car (pg per cell)	0	0	0	0	0
β‐car	0.34 ± 0.11	1.63 ± 0.78	6.44 ± 3.91	10.66 ± 5.35	38.13 ± 7.09
β‐car (pg per cell)	0.04 ± 0.01	0.04 ± 0.02	0.04 ± 0.02	0.03 ± 0.01	0.02 ± 0.01
Diato:Diadino	0	3.26 ± 0.53	0.08 ± 0.01	5.38 ± 0.74	0.57 ± 0.64
PPC:PSC1	0.16 ± 0.01	0.77 ± 0.01	0.15 ± 0.01	0.72	0.23 ± 0.07
PPC:Chl *a*	0.10 ± 0.01	0.35 ± 0.01	0.08	0.35 ± 0.01	0.12 ± 0.03

*Note*: For each pigment, the mean and standard deviation of the absolute concentration (mg · m^−3^) and the concentration per cell (pg · m^−3^ · cell^−1^) is presented. Standard deviations <0.01 mg · m^−3^ are not shown.

Abbreviations: Allo, alloxanthin; Chl *a*, chlorophyll *a*; Chl *c*_1_, chlorophyll *c*_1_; Chl *c*_2_, chlorophyll *c*_2_; Croco, crocoxanthin; Diadino, diadinoxanthin; Diato, diatoxanthin; Fuco, fucoxanthin; PPC, photoprotective carotenoids; PSC, photosynthetic carotenoids; α‐car, α‐carotene; β‐car, β‐carotene.

Macronutrient uptake mirrored the pattern seen in biomass, except during the first LL stage when ~200 μM of DIN was rapidly consumed despite limited cellular growth, likely reflecting lag phase nitrogen assimilation and luxury nitrate storage prior to the exponential growth phase (Figure 2e; Table 1). By Day 24, the initial DIN available (842 μM) had been reduced to 13% in *Porosira glacialis*, 33% in *Fragilariopsis cylindrus*, and 48% in *Geminigera cryophila*. Unlike DIN, almost all phosphate was consumed by the end of the experiment, especially for both diatoms (<2%; <1 μM remaining). Similarly, silicic acid was almost fully depleted at this stage (<4%; <7 μM remaining). On average, the highest nutrient consumption rates were observed in both LL stages, from days 1 to 7 (−37 μM · d^−1^) and from days 17 to 24 (−40 μM · d^−1^; Figure 2f).

### Pigment composition and photoacclimation

*Porosira glacialis* and *Fragilariopsis cylindrus* shared the same pigment suite, although the pennate diatom exhibited substantially lower overall cellular content (e.g., fucoxanthin = 5.53 pg · cell^−1^ vs 0.46 pg · cell^−1^, respectively; Table 2). *Geminigera cryophila*, differed from both diatoms by lacking fucoxanthin, Chl *c*_1_, diadinoxanthin, diatoxanthin, and β‐carotene and by including alloxanthin, crocoxanthin, and α‐carotene (Table 2).

**FIGURE 3:**
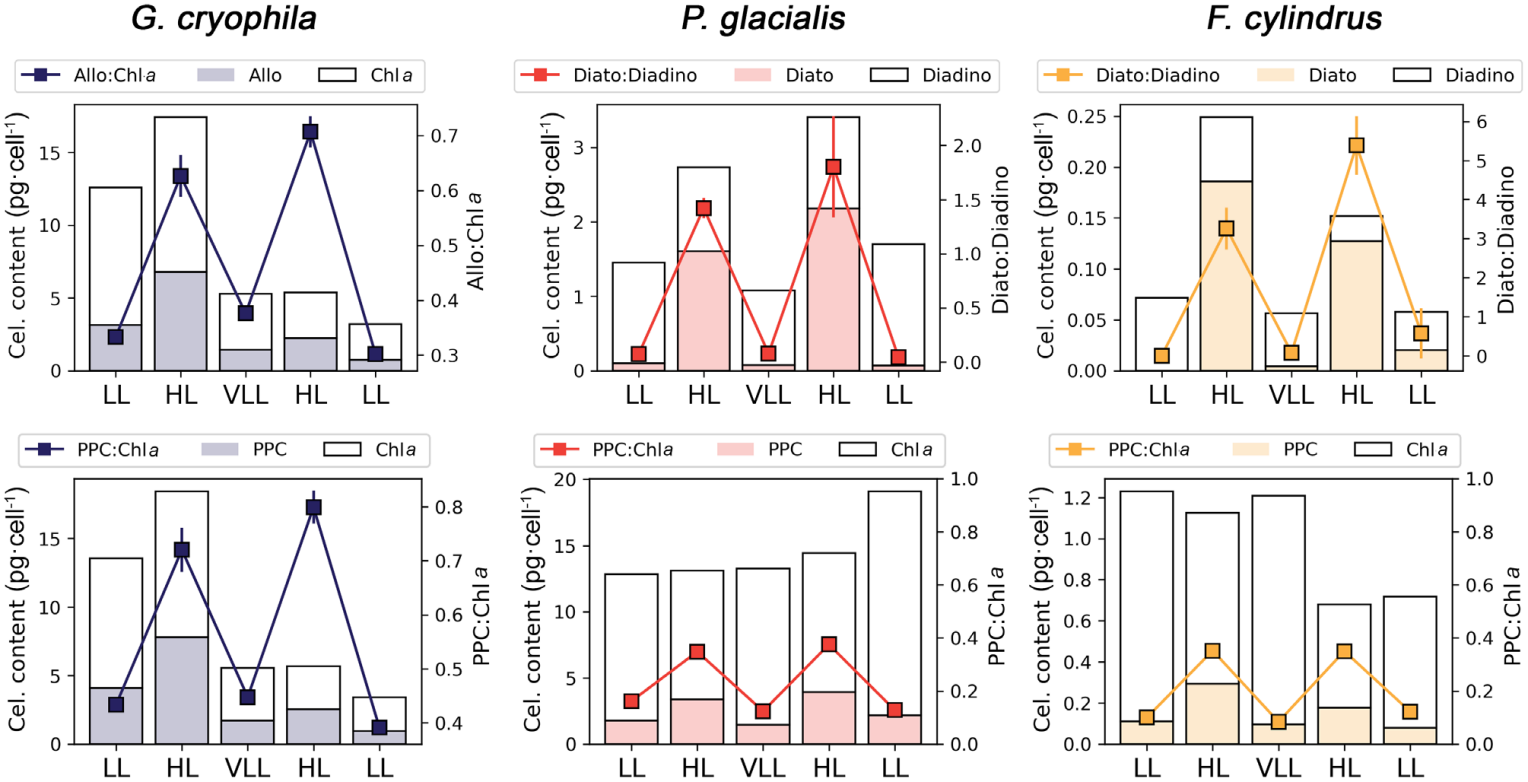
Pigment photoacclimation indices for each light phase of the experiment. Lines in the upper row represent the alloxanthin:chlorophyll *a* ratio (Allo:Chl *a*) for *Geminigera cryophila* (GEM) and the diatoxanthin: diadinoxanthin ratio (Dt:Dd) for both *Porosira glacialis* (POR) and *Fragilariopsis cylindrus* (FRA). Lines in the bottom row show the ratio between photoprotective carotenoids and chlorophyll *a* (PPC:Chl *a*) for all species. Each value represents the mean ± standard deviation of three replicates (*n* = 3). Statistically significant differences among the species were tested using Kruskal–Wallis and Mann–Whitney tests. For each panel, bars present each pigment's cellular content (pg · cell^−1^) proportion.

Shifts in irradiance phases triggered rapid pigment changes in all species, showcasing efficient photoacclimation (Figure 3; Table 2). As such, hierarchical clustering clearly separated samples under HL from samples under LL and VLL for all species (Figure S4). During each HL phase, the PPC:Chl *a* and PPC:PSC ratios were significantly higher than under LL (Table 2; Figure S5; PPC:Chl *a* HL vs. LL: Mann–Whitney *U* = 247, *p* < 0.01; PPC:PSC HL vs. LL: Mann–Whitney *U* = 144, *p* < 0.01). Diatoxanthin increased over 10‐fold under HL in both *Porosira glacialis* (LL and VLL average = 0.08 pg · cell^−1^; HL average = 1.89 pg · cell^−1^) and *Fragilariopsis cylindrus* (LL and VLL average = 0.01 pg · cell^−1^; HL average = 0.16 pg · cell^−1^), a pattern reflected in higher Dt:Dd ratios (Figure 3).

Unlike the two diatoms, *Geminigera cryophila* exhibited a less consistent pigmentary response to light changes throughout the experiment (Figure 3; Table 2). During the first HL stage, the cryptophyte greatly increased its cellular content of Chl *a* (+116%) and alloxanthin (+216%), raising PPC:Chl *a* from 0.43 ± 0.02 to 0.72 ± 0.04 (Wilcoxon, *p* = 0.02; Table 2). However, after the first HL stage, *G*. *cryophila*'s cellular Chl *a* content declined regardless of irradiance, although adjustments in PPC:Chl *a* were still observed according to the incident light (VLL: 0.45 ± <0.01; HL: 0.80 ± 0.03; LL: 0.39 ± 0.01). Therefore, unlike the diatoms, which modulated both photosynthetic and photoprotective pigments according to changes in irradiance, the cryptophyte relied mainly on adjusting its carotenoid content, especially alloxanthin.

### Photosynthetic efficiency (*F*_v_/*F*_m_)

For all species, the maximum PSII quantum yield (*F*_v_/*F*_m_) declined sharply under HL but recovered under LL (Figure S6; Table 1). Across taxa, mean *F*_v_/*F*_m_ was 0.62 ± 0.12 in VLL and 0.55 ± 0.12 in LL, both significantly higher than the 0.31 ± 0.09 recorded under HL (Figure S6; LL vs. HL: Mann–Whitney *U* = 1127, *p* < 0.01; VLL vs. HL: Mann–Whitney *U* = 446, *p* < 0.01).

In low light conditions, a Wilcoxon test showed *Geminigera cryophila* exhibited the highest *F*_v_/*F*_m_ (0.64 ± 0.04), surpassing both the centric diatom *Porosira glacialis* (0.51 ± 0.13; Mann–Whitney *U* = 170.5, *p* < 0.05) and the pennate diatom *Fragilariopsis cylindrus* (0.5 ± 0.12; Mann–Whitney *U* = 196, *p* < 0.01). Under VLL, the *F*_v_/*F*_m_ measured among species converged (0.60–0.65) with no significant interspecific difference. During HL, however, *G. cryophila* and *P. glacialis* maintained similar *F*_v_/*F*_m_ (0.36 ± 0.06 and 0.36 ± 0.04, respectively), whereas *F. cylindrus* dropped to 0.20 ± 0.04 (*G. cryophila* vs. *F. cylindrus*: Mann–Whitney *U* = 80, *p* < 0.01; *P. glacialis* vs. *F. cylindrus*: Mann–Whitney *U* = 81, *p* < 0.01).

### Short‐term light‐stress response

**FIGURE 4:**
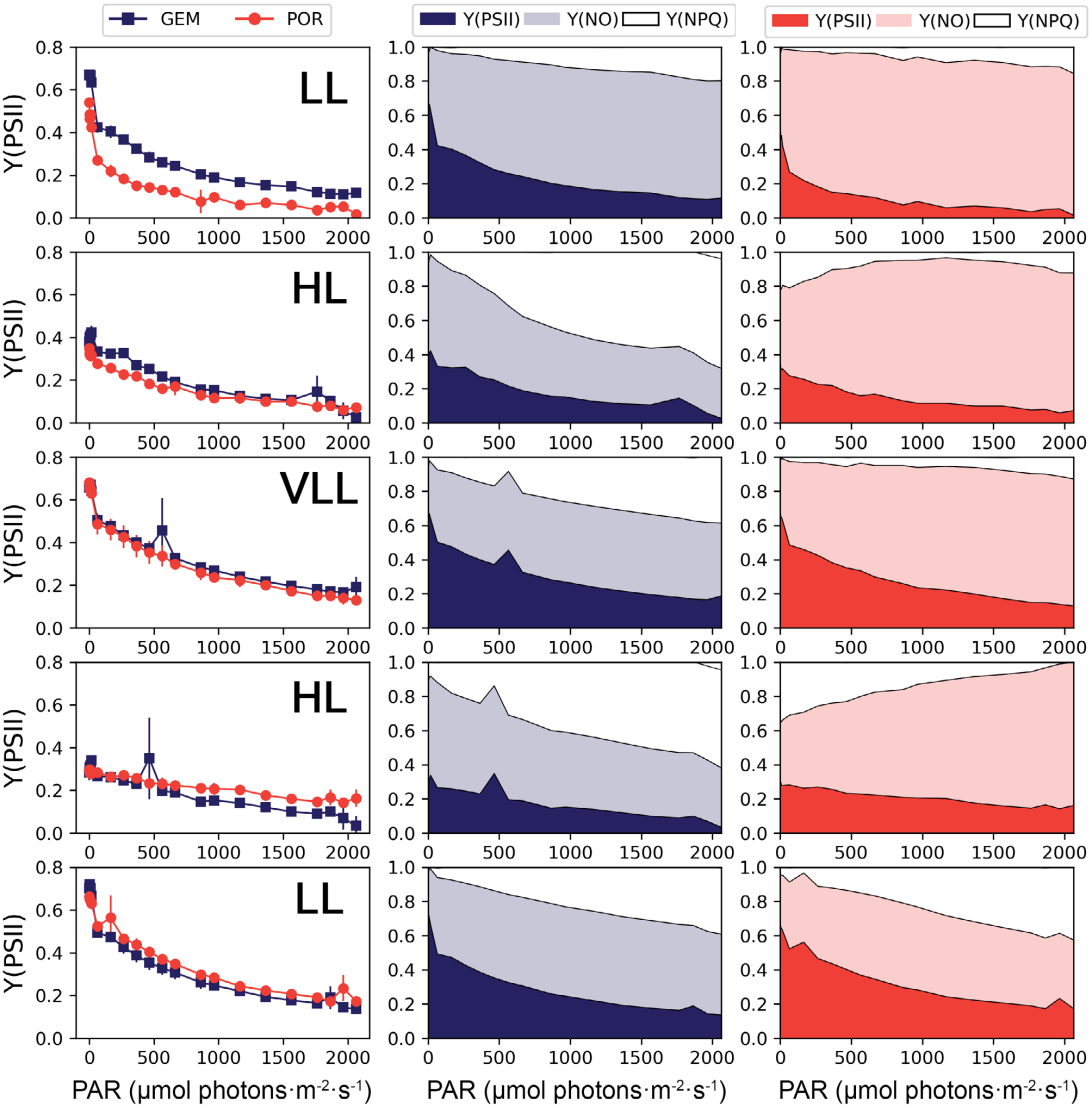
Measurements of the quantum yields of PSII, Y(PSII), energy dissipated through non‐photochemical quenching, Y(NPQ), and energy dissipated through nonregulated processes, Y(NO) across the 21 irradiance steps of the rapid light curves (RLCs; PAR, μmol photons · m^−2^ · s^−1^) conducted at the end of each light phase (from top to bottom: Day 7, Day 11, Day 14, Day 17, and Day 24). The left panels show the comparison of Y(PSII) between *Geminigera cryophila* (GEM) and *Porosira glacialis* (POR), while the middle and right panels illustrate the partitioning of excitation energy into Y(PSII), Y(NPQ), and Y(NO) throughout the RLC for each species. Each value in the Y(PSII) panel (left) represents the mean ± standard of three replicates (*n* = 3). Only the mean is shown for the middle and right panels. Statistically significant differences among the species were tested using Mann–Whitney tests. Note that Y(PSII) + Y(NPQ) + Y(NO) = 1.

**FIGURE 5:**
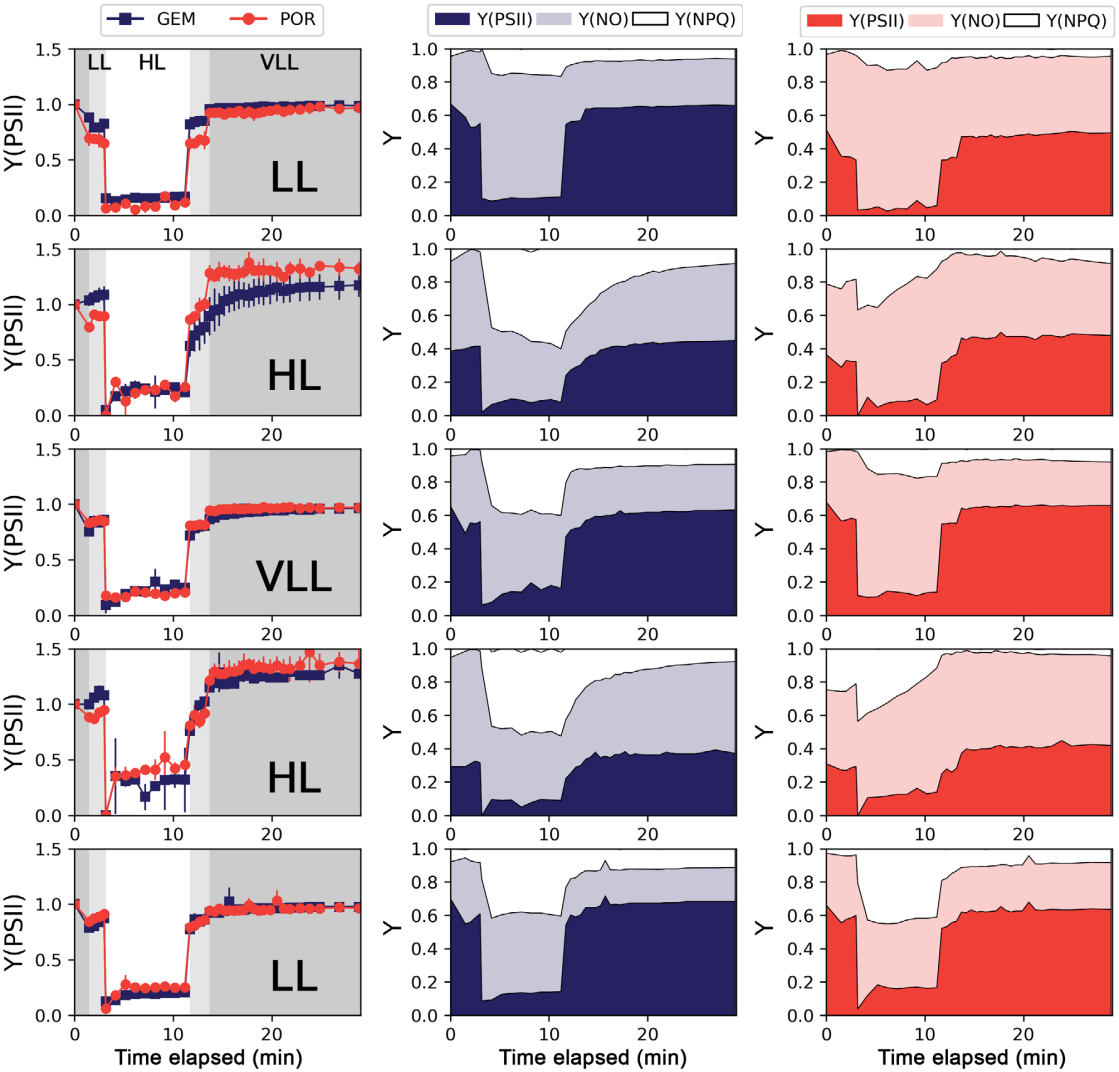
Measurements of the quantum yields of PSII, Y(PSII), of the energy dissipated through non‐photochemical quenching, Y(NPQ), and of the energy dissipated through nonregulated method, Y(NO), over time (minutes) in the light stress induction and recovery experiments (LSREs) conducted at the end of each light phase (from top to bottom: Day 7, Day 11, Day 14, Day 17, and Day 24). The left panels represent the comparison of Y(PSII) between *Geminigera cryophila* (GEM) and *Porosira glacialis* (POR), whereas the middle and right panels exhibit the changes in the fractions of Y(PSII), Y(NPQ), and Y(NO) throughout the RLC for each species. Each value in the Y(PSII) panel (left) represents the mean ± standard of three replicates (*n* = 3). Only the mean is shown for the middle and right panel. Statistically significant differences among the species were tested using Mann–Whitney tests. Note that Y(PSII) in the left panel has been standardized to the initial Y(PSII) (*F*_v_/*F*_m_) and that Y(PSII) + Y(NPQ) + Y(NO) = 1.

The RLC and LSRE results showed clear interspecific differences (Figures 4 and 5). Nevertheless, *Fragilariopsis cylindrus* measurements were too noisy (due to its tendency to form aggregates) to maintain a homogeneous sample within the Phyto‐PAM stirred chamber, lowering accuracy. As such, only *Geminigera cryophila* and *Porosira glacialis* results are presented here. (Full *F. cylindrus* data is in Figures S7–S10).

The RLCs highlighted a stronger regulated quenching capacity in the cryptophyte (Figure 4; Table 2). Although Y(PSII) decreased with the increasing irradiance on each RLC step similarly across both species, different patterns of excess energy dissipation were observed. At the end of the RLCs (688 μmol photons · m^−2^ · s^−1^), *Geminigera cryophila* reached an average Y(NPQ) of 0.44 ± 0.17 compared to the much lower value of 0.17 ± 0.15 for *Porosira glacialis*. This difference in Y(NPQ) was particularly evident in the RLCs run during the HL phases (*G. cryophila*: 0.61 ± 0.06 vs. *P*. *glacialis*: 0.06 ± 0.08; Mann–Whitney *U* = 36, *p* < 0.01). Under HL, *P. glacialis* instead dissipated much of its excess energy via nonregulated pathways: final Y(NO) = 0.82 ± 0.05.

The parameters derived from the RLC further supported the existence of patterns in light use (Table 3). For *Geminigera cryophila*, Chl *a* remained relatively high across all phases (1.35 ± 0.26), while ETRmax peaked during the VLL and the second LL stages (323.26 ± 12.56 and 289.32 ± 32.65, respectively). In contrast, *Porosira glacialis* exhibited lower ɑ during HL (0.84 ± 0.09) and higher values under VLL (1.52 ± 0.17) and in the second LL stage (1.7 ± 0.11). Furthermore, its ETRmax more than doubled from HL1 (147.62 ± 12.17) to HL2 (293.13 ± 20.47) and reached 347.66 ± 29.35 at the final LL stage. Under HL conditions, the centric diatom also exhibited higher average *E*k (262.61 ± 81.3) compared to the cryptophyte (158.26 ± 44.83), showcasing a higher light saturation for *P. glacialis* under high irradiance. *Geminigera cryophila* had stable ɑ throughout different light levels, indicating that it was able to rapidly and efficiently capture energy across a wide light range, whereas *P. glacialis* lowered its ɑ and raised *E*k under HL, suggesting slower, yet efficient adjustments as seen by its high ETRmax.

**TABLE 3:** Parameters extracted from the rapid light curves (RLCs) conducted at the end of each light phase for both *Geminigera cryophila* (GEM) and *Porosira glacialis* (POR).

Species	Parameters	LL (D7)	HL (D11)	VLL (D14)	HL (D17)	LL (D24)
GEM	α	1.32 ± 0.19	1.16 ± 0.13	1.55 ± 0.02	1.12 ± 0.39	1.58 ± 0.15
	rETRmax	211 ± 33	167 ± 14	323 ± 15	176 ± 22	290 ± 39
	*E*k	161 ± 37	146 ± 29	208 ± 12	171 ± 69	183 ± 11
	Y(PSII)_INIT_	0.67 ± 0.01	0.38 ± 0.02	0.66 ± 0.01	0.28 ± 0.04	0.7 ± 0.01
	Y(PSII)_FINAL_	0.12 ± 0.01	0.03 ± 0.04	0.19 ± 0.05	0.03 ± 0.05	0.14 ± 0.02
	Y(NPQ)_INIT_	0.04 ± 0.01	0.07 ± 0.01	0.07 ± 0.03	0.12 ± 0.1	0.03
	Y(NPQ)_FINAL_	0.20 ± 0.04	0.64 ± 0.02	0.39 ± 0.13	0.57 ± 0.07	0.39 ± 0.02
	Y(NO)_INIT_	0.29 ± 0.01	0.55 ± 0.01	0.28 ± 0.01	0.59 ± 0.11	0.27 ± 0.01
	Y(NO)_FINAL_	0.69 ± 0.05	0.29 ± 0.02	0.43 ± 0.10	0.35 ± 0.07	0.47 ± 0.04
POR	ɑ	0.71 ± 0.07	0.78 ± 0.06	1.52 ± 0.21	0.89 ± 0.11	1.70 ± 0.13
	rETRmax	96 ± 27	148 ± 14	282 ± 33	296 ± 29	351 ± 36
	*E*k	137 ± 46	189 ± 6	186 ± 9	336 ± 59	204 ± 9
	Y(PSII)_INIT_	0.54 ± 0.02	0.35 ± 0.02	0.68 ± 0.02	0.30 ± 0.03	0.66 ± 0.01
	Y(PSII)_FINAL_	0.02 ± 0.01	0.07 ± 0.01	0.13 ± 0.01	0.16 ± 0.04	0.17 ± 0.02
	Y(NPQ)_INIT_	0.01 ± 0.01	0.20 ± 0.11	0.02 ± 0.00	0.34 ± 0.15	0.05 ± 0.06
	Y(NPQ)_FINAL_	0.16 ± 0.06	0.12 ± 0.09	0.12 ± 0.03	0	0.43 ± 0.07
	Y(NO)_INIT_	0.44 ± 0.02	0.45 ± 0.12	0.30 ± 0.02	0.36 ± 0.12	0.29 ± 0.06
	Y(NO)_FINAL_	0.83 ± 0.04	0.81 ± 0.08	0.74 ± 0.02	0.84 ± 0.05	0.40 ± 0.06

*Note*: For more details on each parameter, please consult Section 2.3.2.

Abbreviations: HL, High light; LL, Low light; VLL, Very low light.

The LSREs confirmed faster and more flexible NPQ by *Geminigera cryophila* (Figure 5). Both *G. cryophila* and *Porosira glacialis* significantly reduced Y(PSII) within the 9‐min HL stress period of the LSRE (PAR = 650 μmol photons · m^−2^ · s^−1^), yet the cryptophyte raised and maintained its Y(NPQ), whereas the diatom dissipated nearly all its excess energy through nonregulated pathways, that is, Y(NO). For instance, in the HL stages, by the end of the stress period of the LSRE (~11 min), *G. cryophila* exhibited >50% Y(NPQ) whereas *P. glacialis* only exhibited ~12% Y(NPQ). Upon the return to LL, *G. cryophila* was also able to regulate its Y(NPQ) in a slower, more relaxed way than *P. glacialis*. The only exception was observed in the last LSRE (day 24), where both species exhibited very similar recovery patterns.

### In situ environmental preferences

**FIGURE 6:**
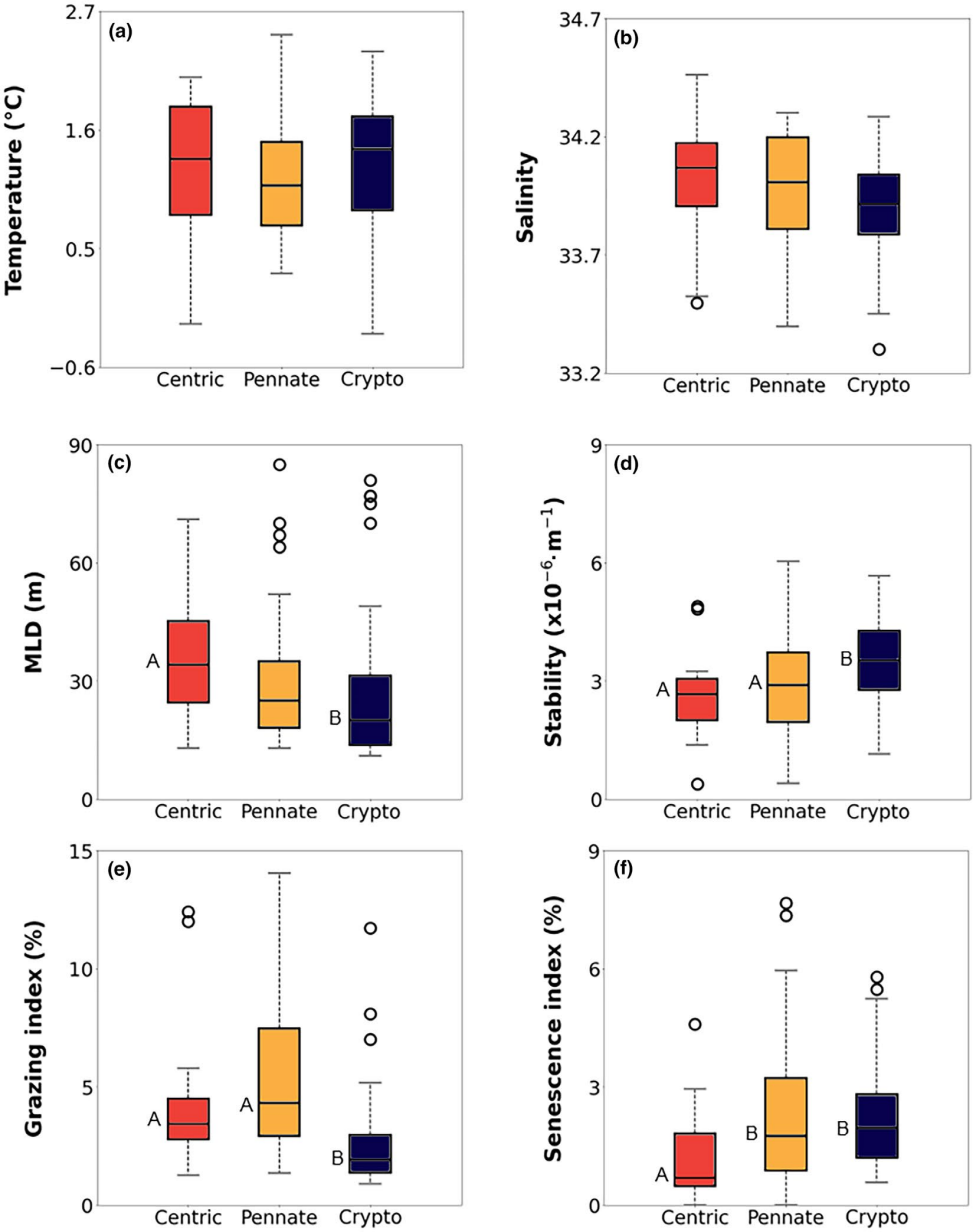
Comparison of seawater temperature (a) and salinity (b), mixed layer depth (MLD; m; c), water column stability (×10^−6^ · m^−1^; d), grazing index (%; e), and senescence index (%; f) between stations with phytoplankton communities dominated by centric diatoms (red), pennate diatoms (yellow) or cryptophytes (blue). Community dominance here refers to either >50% of total Chl *a* or total cell abundance (see Section 2.4). The capital letters indicate statistically distinct groups based on pairwise multiple comparisons using Dunn's Method following a Kruskal–Wallis test (*p* < 0.05). Groups sharing the same letter are not significantly different.

Pigment and cell abundance data from 176 coastal WAP stations allows to identify 53 samples dominated by cryptophytes and 123 samples dominated by diatoms (centric‐dominated = 17; pennate‐dominate = 42; remainder were excluded since no cell abundance data were available). In terms of environmental conditions (Figure 6), cryptophytes dominated in shallower mixed layers (MLD = 26.5 ± 18.4 m) compared to centric diatoms (MLD = 38 ± 23.3 m; Mann–Whitney *U* = 234.5, *p* < 0.01), whereas pennate diatom occupied intermediate mixed layers. Water column stability also followed the same pattern, as cryptophytes favored the most stratified stations (Kruskal–Wallis *H*: 11.05, *p* < 0.01). Both sea surface temperature and salinity did not statistically differ among groups, averaging 1.23 ± 0.58°C and 33.94 ± 0.24°C, respectively.

Additionally, both the grazing and senescence indexes also hinted at differences between groups. The grazing index (signaling higher grazing pressure) was twice as high in diatom‐dominated communities (5.21% ± 3.47%) than in cryptophyte‐dominated ones (2.54% ± 1.96%; Kruskal–Wallis *H*: 33.0, *p* < 0.01). The senescence index, in contrast, was significantly higher in samples dominated by pennate diatoms and cryptophytes (typical of opportunistic groups), indicating that phytoplankton cell deterioration was lower when centric diatoms were more abundant.

## DISCUSSION

The 24‐day experiment reproduced the day‐to‐week irradiance swings that coastal waters of the WAP experience during summer stratification. All three species—*Geminigera cryophila*, *Fragiliariopsis cylindrus*, and *Porosira glacialis*—successfully grew through every phase, confirming the capacity of Antarctic phytoplankton to withstand rapid transitions from very low (<10 μmol photons · m^−2^ · s^−1^) to high irradiance (>500 μmol photons · m^−2^ · s^−1^; Table 1).

### Fast photoacclimation to changes in light

Our results indicate that Antarctic phytoplankton can efficiently adjust their pigment machinery in response to rapid changes in irradiance, providing insight into how these taxa successfully grow in highly variable Antarctic surface waters. All three species modified their Chl *a* content and adjusted photoprotective pigments when transitioning between light regimes (Figure 3). The ability to rapidly acclimate within only 3 days when shifting between HL (400–500 μmol photons · m^−2^ · s^−1^) and VLL (6 μmol photons · m^−2^ · s^−1^), and vice versa, shows a high degree of physiological flexibility in response to fluctuating light availability.

Despite this common capacity for rapid photoacclimation, the three species appeared to exhibit distinct growth responses to the imposed sequence of light regimes. The smaller sized pennate diatom *Fragilariopsis cylindrus* and the cryptophyte *Geminigera cryophila* both increased in cell abundance following the transition from LL to HL, with an even stronger increase during the subsequent shift from HL to VLL. In contrast, growth declined when irradiance increased again from VLL to HL, before recovering during the final LL phase. The large centric diatom *Porosira glacialis* exhibited a different trajectory, with cell abundance increasing progressively from LL to HL and VLL but showing little additional change during the final transitions from VLL to HL and back to LL (Figure 2, Table 1). These contrasting growth patterns provided an important framework for interpreting the species‐specific photoacclimation strategies observed, despite all taxa achieving overall growth success by the end of the experiment. It is also important to note that results from the initial LL phase should be interpreted with caution when comparing species, as early growth responses can be strongly influenced by differences arising from the pre‐acclimation period and from initial cell abundances at Day 0.

Previous studies reported mixed responses to fluctuating light in Antarctic phytoplankton. Kropuenske et al. (2010) observed rapid photoacclimation within 4 days in cultures of *Phaeocystis antarctica* and *Fragilariopsis cylindrus*, whereas Viljoen et al. (2018) observed limited pigment acclimation in a diatom‐dominated natural assemblage even after 6 days under contrasting light levels. Moreover, it has often been assumed that acclimation to LL is slower than acclimation to HL (Dubinsky & Stambler, 2009; Post et al., 1984). However, our results do not support this assumption, since all three species rapidly photoacclimated following decreases in light intensity (LL and VLL), exhibiting a short photoacclimation period comparable to that observed after HL exposure, showing a high degree of photoacclimative flexibility (Figure 3).

For both diatoms, the most evident sign of photoacclimation was the increase of the xanthophyll pool per cell and the concomitant increase in the conversion of diadinoxanthin to diatoxanthin under HL conditions (Figure 3). In the diatom xanthophyll cycle, diadinoxanthin is enzymatically de‐epoxidated and converted to diatoxanthin under HL (Falkowski & Raven, 2013). Diatoxanthin acts as a quencher of excitation energy within the PSII antenna, facilitating heat dissipation under HL (Falkowski & Raven, 2013). This process can occur very rapidly (within minutes), unlike changes in Chl *a* or other structural pigments, which take longer (Olaizola et al., 1994). The xanthophyll cycle is therefore crucial in allowing diatoms to acclimate to HL environments, such as those near the ice edges during the austral summer (Hashihama et al., 2010), and in allowing both *Fragilariopsis cylindrus* and *Porosira glacialis* to grow successfully throughout the experiment. Interestingly, *F. cylindrus* exhibited Dt:Dd ratios over twice as high as *P. glacialis* did under HL. Although this could suggest that *F. cylindrus* might have a better capacity of synthesizing diatoxanthin and dissipating excess energy more efficiently through the diatoxanthin cycle, it is important to note that the much larger biovolume of *P. glacialis* could lead to higher shelf‐shading, which would reduce the need to synthesize as much diatoxanthin as a smaller cell (Dubinsky & Stambler, 2009).

*Geminigera cryophila*, unlike both diatoms, lacks a xanthophyll cycle, and its photoacclimation was characterized by an increase in the Allo:Chl *a* ratio under HL conditions, reinforcing alloxanthin's key role as a photoprotective pigment. The average Allo:Chl *a* observed under HL (0.66 ± 0.05) closely matched the Allo:Chl *a* values reported by Mendes et al. (2023) after subjecting the same *G. cryophila* strain to 536 μmol photons · m^−2^ · s^−1^ (Allo:Chl *a* = 0.66). Similarly, the lower Allo:Chl *a* ratio (0.34 ± 0.03) observed under LL and VLL aligned with previous observations by Cunningham et al. (2019) and Mendes et al. (2023; Allo:Chl *a* = 0.48 and 0.3, respectively). Notably, previous studies conducted under constant light conditions interpreted increases in Allo:Chl *a* primarily as a consequence of reduced cellular Chl *a*, consistent with a light‐harvesting role of alloxanthin (e.g., Mendes et al., 2023; Trimborn et al., 2019). In contrast, the fluctuating light regime used in the present study may promote a more photoprotective function of alloxanthin, raising the need for future experiments explicitly comparing constant and fluctuating light regimes to resolve the role of alloxanthin in cryptophyte photoacclimation.

Although not measured in this study, phycobiliproteins, particularly phycoerythrin, may have played a significant role in light harvesting during the second half of the experiment, when both Chl *a* and alloxanthin declined. Previous work showed that *Geminigera cryophila* exhibits high phycoerythrin:Chl *a* ratios (2–3.5), especially under LL conditions (Mendes et al., 2023). The high nitrate consumption observed in the final LL phase could also be linked to nitrogen‐rich phycobiliproteins contributing to efficient light harvesting under low irradiance, thereby sustaining high cell growth toward the end of the treatment.

### Size‐dependent growth and nutrient consumption in the two diatoms

Light availability is often considered a crucial factor influencing phytoplankton growth rates. In general, growth increases almost linearly at low irradiance until it saturates at optimal light levels and then subsequently declines due to photoinhibition (Edwards et al., 2015; Langdon, 1988; Talmy et al., 2013). Very low light can prevent growth altogether (Kiefer & Cullen, 1991; Lee et al., 2022). In addition, interspecific differences can also play important roles in shaping the growth of phytoplankton communities under variable light intensities (Edwards et al., 2016). In this study, however, there was no clear evidence of light‐limited growth in either LL or HL stages, again reinforcing Antarctic phytoplankton's high capability to endure this complex light environment.

The average and maximum μ observed in this study were in line with those calculated for Antarctic phytoplankton in previous works (e.g., Gleitz et al., 1996; Lee et al., 2022; Mosby & Smith Jr, 2015; Trimborn et al., 2019). Although these growth rates might seem relatively low compared to those when in temperate waters, it is crucial to consider the impact of the low temperatures typical of polar waters in significantly limiting phytoplankton growth rates (Edwards et al., 2016).

The observed differences in maximum growth rates observed between diatoms *Fragilariopsis cylindrus* (0.5 · d^−1^) and *Porosira glacialis* (0.21 · d^−1^) are consistent with those reported between small pennate and large centric diatoms living in the Amundsen Sea (0.42 · d^−1^ and 0.16 · d^−1^, respectively; Lee et al., 2022). For cryptophyte *Geminigera cryophila*, the average growth rates in this study (0.19 ± 0.02 · d^−1^) were also comparable to those reported by Trimborn et al. (2019) and Mendes et al. (2023) for the same species, with cultures established from the WAP (0.08–0.23 · d^−1^ and 0.21–0.24 · d^−1^, respectively). Interestingly, some of the highest maximum growth rates were observed under VLL, contrasting with Lee et al. (2022) and Kiefer and Cullen (1991), who reported very low (<0.1 · d^−1^) and negative growth rates in Antarctic species under LL conditions (i.e., 9 μmol photons · m^−2^ · s^−1^ in Kiefer & Cullen, 1991).

The major interspecific differences in terms of growth and biomass were observed between the diatoms. Each species employed different growth strategies throughout the treatment, thereby supporting the hypothesis that centric and pennate diatoms occupy distinct niches in the WAP (Costa et al., 2023). Although *Porosira glacialis* had lower cell abundances than *Fragilariopsis cylindrus*, its larger cell size resulted in higher biovolume toward the end of the experiment. In contrast, cellular Chl *a* content declined in *F. cylindrus* (and *G. cryophila*) at Day 24, whereas it increased in the large centric diatom. Furthermore, although *F. cylindrus* exhibited a higher maximum growth rate, its growth was not as consistent over time as that of *P. glacialis*. As a result, although the mean growth rates of the two diatoms differed significantly, their average growth rates measured at the end of the experiment were nearly identical (*P. glacialis*: 0.20 · d^−1^; *F. cylindrus*: 0.23 · d^−1^). Furthermore, *P. glacialis* also consumed significantly more DIN compared to *F. cylindrus*, which consumed slightly more phosphate and silicic acid by the end of the experiment (Table 1).

Overall, the differences observed between *Porosira glacialis* and *Fragilariopsis cylindrus* resembles those between K‐ and r‐strategists (MacArthur & Wilson, 1967). The larger *P. glacialis* exhibited slower but consistent growth over time with minimal fluctuations between light phases and more consumption of available DIN. In contrast, the much smaller *F. cylindrus* favored rapid growth and likely had a lower half‐saturation constant for nitrate uptake given its much lower cell volume (55.2 μm^3^ compared to *P. glacialis* at 10,879 μm^3^). Despite the large difference in DIN consumption, both species exhibited a similar nitrogen‐to‐chlorophyll yield (*q*), that is, the amount of Chl *a* that was produced from the amount of DIN consumed, from Day 7 to Day 24 (*q*_*P*. *glacialis*_ = 3.39 mg Chl *a* · μM^−1^; q_*F*. *cylindrus*_ = 3.85 mg Chla *a* · μM^−1^; calculated following Gowen et al., 1992). This suggests that a significant portion of the DIN consumed by *P. glacialis* was not converted to biomass and was instead, likely, stored in large vacuoles, a strategy often employed by larger diatoms during periods of high nutrient availability (Behrenfeld et al., 2021).

Although it was not possible to perform a more in‐depth study of the photophysiological parameters of *Fragilariopsis cylindrus* due to the low accuracy of the RLC and LRSE results, the species appeared to rapidly and efficiently handle changes to HL conditions. A previous study comparing a *F. cylindrus* strain from the Ross Sea with *Phaeocystis antarctica*, also showed that *F. cylindrus* was significantly less susceptible to photoinhibition (Mills et al., 2010). This result matched previous findings by Costa et al. (2023) and Nardelli et al. (2023), who reported that pennate diatoms tended to exhibit a more opportunistic ecological behavior and could thrive in regions characterized by shallower mixed layers and with higher sea ice melt during the peak of summer, when light availability is at its maximum (Costa et al., 2020, 2023; Nardelli et al., 2023; Pan et al., 2020; Petrou et al., 2016).

As regional atmospheric and ocean warming continues, coupled with increased ice melt and intensified stratification along the coastal WAP, it is important to consider how a potential shift from centric to pennate diatoms may affect the WAP marine ecosystem. Larger, centric diatoms, such as *Porosira glacialis*, are often associated with deeper mixed layers, where they form high biomass blooms and contribute substantially to oceanic CO_2_ uptake (Brown et al., 2019; Costa et al., 2020, 2021). Moreover, the Antarctic krill (*Euphausia superba*), a key species in the Antarctic food web, preferentially feed on larger centric diatoms (Haberman et al., 2003; Pauli et al., 2021; Quetin & Ross, 1985). Hence, this potential shift could have significant implications for the WAP.

### Cryptophytes: An emergent, resilient group

Cryptophytes are another important phytoplankton group expected to become increasingly more abundant along the coastal WAP in the near future (Mendes et al., 2023). Over the past 2 decades, several studies have reported cryptophytes outgrowing diatoms and other phytoplankton groups in areas affected by sea ice and glacial melting (Brown et al., 2019; Costa et al., 2023; Moline et al., 2004; Mendes et al., 2013, 2018, 2023; Nardelli et al., 2023; Schofield et al., 2017). Although the exact factors driving this recent ecological success of cryptophytes are not yet fully understood, a recent study showed that *Geminigera cryophila* is highly flexible and efficient in rapidly photoregulating changes to HL conditions, allowing it to thrive under the HL stress typically associated with stratified shallow upper layers (Mendes et al., 2023).

Ours results confirm the ability of cryptophytes to endure high light conditions thanks to their highly efficient NPQ. The contrasting NPQ patterns observed during the RLCs (Figure 4), including high Y(NPQ) at low irradiance and non‐monotonic responses with increasing light, highlighted species‐specific differences in photoprotective regulation between diatoms and cryptophytes. Following sustained HL exposure, *Geminigera cryophila* exhibited a rapid relaxation and reengagement of NPQ, whereas *Porosira glacialis* retained elevated NPQ after dark acclimation but showed a more limited capacity to further increase energy dissipation during subsequent light steps. In addition, *G. cryophila* was able to maintain high Y(NPQ) during prolonged periods of high irradiance (Figure 5). The key exception occurred at the end of the experiment (Day 24; Figure 4), when the comparatively improved HL response of *P. glacialis* may partly reflect self‐shading effects associated with the high cell densities and large cell size attained during the final LL phase, effectively reducing the irradiance experienced by individual cells.

Nonphotochemical quenching is an essential process for protecting phytoplankton cells from over‐excitation in HL environments (Kaňa et al., 2012). Prolonged overexcitation can produce harmful reactive oxygen species (ROS), which are detrimental to the cell's pigments, proteins, and lipids (Li et al., 2009). However, since *Porosira glacialis* managed to grow steadily over the 24 days, it does not appear overexcitation impacted its growth at a larger temporal scale (days–weeks). It is possible that the centric diatom may efficiently scavenge ROS and repair the D1 and D2 reaction centers of the PSII, preventing photoinhibition. Furthermore, *P. glacialis* contains mycosporine‐like amino acids (MAAs), compounds that have important photoprotective roles by protecting intracellular organs against ultraviolet radiation (Ha et al., 2014), which could be an advantage when exposed to strong sunlight.

The mechanism of NPQ in cryptophytes differs significantly from that in diatoms (Kaňa et al., 2012). Firstly, although diatoms utilize the diatoxanthin cycle in NPQ (Dubinsky & Stambler, 2009), cryptophytes lack a xanthophyll cycle. Secondly, cryptophytes exhibit a very fast and flexible NPQ more akin to the quenching observed in higher plants (Kaňa et al., 2012). Thirdly, NPQ in cryptophytes involves direct protonation of antennae, again similar to higher plants (Corbella et al., 2018). Furthermore, phycobiliproteins play a crucial role in light harvesting in cryptophytes, but Kaňa et al. (2012) suggested that NPQ does not occur directly in the phycobiliproteins, meaning that any excess radiation captured by phycobiliproteins must be swiftly transferred to chlorophyll *a*/*c* antennae of PSII for dissipation.

Although Kaňa et al. (2012) suggested that prolonged exposure to excessive irradiation may potentially lead to photoinhibitory damage to PSII in cryptophytes, due to the additional light captured by phycobiliproteins and the absence of a xanthophyll cycle (Kaňa et al., 2012), damage was not observed in our work. In fact, no signs of photodamage were seen during our experiment, despite two extended periods of HL (7 days in total). This observation was evidenced by: (i) the highest *F*_v_/*F*_m_ was observed at the end of the experiment (Day 24; Figure S4), and (ii) the final Y(PSII) in the LRSE was always close to or slightly higher than the initial Y(PSII). A recent study on *Geminigera cryophila* indicated that prolonged exposure (2–3 consecutive weeks) to HL can significantly reduce growth rates and carbon fixation (Camoying & Trimborn, 2023), suggesting limitations under sustained HL conditions. Interestingly, this negative impact was mitigated when the species was exposed to a slight temperature increase (4°C, +2°C from ambient; Camoying & Trimborn, 2023; Trimborn et al., 2019).

Our results therefore suggest that cryptophytes may have an advantage over diatoms in the short term (hours–days) due to their fast and flexible NPQ, yet nothing indicated that this advantage could be maintained across a longer timescale (weeks). Nevertheless, this short‐term advantage may already be significant given how variable PAR can be in the upper water column due to factors such as cloud cover, snow, sea ice, and rapid stratification caused by meltwater (Lazzara et al., 2007; Mendes et al., 2023; van de Poll et al., 2011). Although the experimental design employed in the present study permitted the isolation of isolated physiological responses to abrupt changes in irradiance, it did not fully reproduce the continuous and diurnally modulated light environment experienced by Antarctic phytoplankton during summer, where gradual day–night transitions are superimposed by stochastic variability linked to these environmental drivers. Consequently, the integration of in situ light observations is essential to place laboratory‐based photoacclimation responses into a realistic environmental context.

The results from our in situ analysis further corroborate the existence of distinct environment niches between diatoms and cryptophytes (Figure 6). Cryptophytes dominate in stable, shallow mixed layers (<25 m) with low grazing pressure, exactly the situation in which its efficient NPQ mechanisms could protect cells from excessive light. Centric diatoms, that is, *Porosira glacialis*, conversely, are associated with higher grazing pressure, which is indicative of diatom's higher vulnerability to grazing. Although grazing was not tested in our experiments, feeding simulations using Antarctic krill and different species of phytoplankton have shown that krill actively select diatoms instead of cryptophytes within a mixed community (Haberman et al., 2003), which is another potential advantage for cryptophytes.

## CONCLUSIONS

This study demonstrated that Antarctic phytoplankton exhibit rapid and flexible photoacclimation responses to abrupt changes in the light available over a 24‐day period, reflecting their capacity to cope with the highly dynamic light regimes characteristic of the Antarctic summer off the WAP. Although both cryptophytes and diatoms were able to grow successfully, our results show that they rely on distinct, species‐specific strategies under fluctuating light conditions.

The cryptophyte *Geminigera cryophila* displayed a particularly flexible short‐term photoprotective response, characterized by rapid modulation of NPQ under HL, which likely provides it a competitive advantage during sustained or sudden HL exposure. In contrast, the diatoms exhibited more constrained photophysiological adjustments yet remained equally successful over the day‐scale time frame assessed in this experiment, with differences between the centric and pennate species reflecting contrasting growth and size‐related strategies. Overall, these results highlight the complexity by which changes in light availability may influence phytoplankton community composition.

Projected climate‐driven changes in the WAP, including reduced sea‐ice cover, enhanced meltwater stratification, and increased cloud variability, are expected to increase both the magnitude and frequency of abrupt light fluctuations experienced by phytoplankton in the upper water column (Ferreira et al., 2020, 2024; Intergovernmental Panel on Climate Change, 2021). Our results suggest that such conditions may favor cryptophytes at short temporal scales (hours). No evident competitive advantages were identified over longer timescales (days–weeks).

Given the important consequences that shifts in phytoplankton composition may have for Southern Ocean food‐web structure and biogeochemical cycling (Brown et al., 2019), future studies must continue to deepen our understanding of the environmental preferences of Antarctic phytoplankton. Regarding light responses, it will be essential to incorporate higher frequency in situ physiological measurements, allowing for more realistic light regimes that account for both diurnal and stochastic variability. The combined use of laboratory experiments, in situ observations, and emerging tools such as hyperspectral satellite ocean‐color sensors, autonomous biooptical platforms, and molecular genomic approaches will be key to advancing our understanding of how Antarctic phytoplankton communities respond to ongoing environmental change.

## AUTHOR CONTRIBUTIONS

**Afonso Ferreira:** Conceptualization (equal); data curation (equal); formal analysis (lead); investigation (equal); methodology (equal); software (equal); visualization (lead); writing – original draft (lead). **Raul R. Costa:** Conceptualization (equal); data curation (equal); formal analysis (equal); investigation (equal); methodology (equal); writing – review and editing (equal). **Bruno Jesus:** Conceptualization (equal); formal analysis (equal); investigation (equal); methodology (equal); software (equal); supervision (equal); visualization (supporting); writing – review and editing (equal). **Ana C. Brito:** Conceptualization (supporting); funding acquisition (lead); investigation (equal); project administration (equal); supervision (lead); writing – review and editing (equal). **Savênia B. Silveira:** Investigation (equal); methodology (equal); resources (equal); writing – review and editing (equal). **Vanda Brotas:** Funding acquisition (equal); project administration (equal); supervision (lead); validation (equal); writing – review and editing (equal). **Catarina V. Guerreiro:** Funding acquisition (equal); project administration (equal); resources (equal); writing – review and editing (equal). **Eduardo Resende Secchi:** Funding acquisition (equal); supervision (equal); writing – review and editing (equal). **Carlos Rafael B. Mendes:** Conceptualization (lead); funding acquisition (lead); investigation (equal); methodology (equal); supervision (lead); validation (equal); writing – review and editing (equal).

## Supporting information


**Figure S1.** Experimental light regime applied during the experiment. Photosynthetically active radiation (PAR; μmol · photons · m^−2^ · s^−1^) is shown as a function of time (days), with diel on:off cycles within each light stage. Shaded areas indicate the successive light treatments: low light (LL), high light (HL), very low light (VLL), followed by a return to HL and LL.
**Figure S2.** Biovolume (μm^3^) variation for *Geminigera cryophila* (blue), *Porosira glacialis* (red), and *Fragilariopsis cylindrus* (yellow) throughout the treatment. Shaded areas in panels (a), (d), and (g) represent the periods of low and very low light.
**Figure S3.** Variation of chlorophyll‐*a* concentration (Chl *a*) in percentage compared to the value measured on Day 7. Shaded areas in panels (a), (d), and (g) represent the periods under of the low (30–40 μmol · photons · m^−2^ · s^−1^) and very low (6 μmol · photons · m^−2^ · s^−1^) light stages. FRA, *Fragilariopsis cylindrus*; GEM, *Geminigera cryophila*; POR, *Porosira glacialis*.
**Figure S4.** Agglomerative hierarchical clustering (Ward's method) of all samples collected during the treatment (*N* = 45) based on their intracellular pigment concentrations. * and° correspond to samples collected during high light phases (HL) and low light and very low light phases (LL + VLL), respectively. CRYP, *Geminigera cryophila*; FRAG, *Fragilariopsis cylindrus*; PORO, *Porosira glacialis*.
**Figure S5.** Boxplot of pigment concentrations in each light phase (LL: low light, HL: high light, and VLL: very low light), showcasing the changes in pigments as part of photoacclimation for *Geminigera cryophila*, *Porosira glacialis*, and *Fragilariopsis cylindrus*. Each pigment's concentration was normalized between 0 and 1.
**Figure S6.** Changes in *F*_v_/*F*_m_ (maximum quantum yield of the PSII) throughout the experiment for each species (a) and the differences observed between different light phases (b). Data from all species were merged for the boxplots in (b). FRA, *Fragilariopsis cylindrus*; GEM, Geminigera *cryophila*; POR, *Porosira glacialis*.
**Figure S7.** Measurements of the quantum yields of PSII, Y(PSII), of the energy dissipated through non‐photochemical quenching, Y(NPQ), and of the energy dissipated through non‐regulated methods, Y(NO), for each step of the rapid light curves (RLCs) run with *Fragilariopsis cylindrus*. The left panels represent the variations of Y(PSII), whereas the middle and right panels exhibit the changes in the fractions of Y(PSII), Y(NPQ), and Y(NO) throughout the RLC (note that Y[PSII] + Y[NPQ] + Y[NO] = 1). Gaps in the panels correspond to invalid measurements caused by very high variability.
**Figure S8.** Measurements of the rETR (relative Electron Transport Rate) for each step of the rapid light curves (RLCs) run with all three species throughout the experiment (Days 7, 11, 14, 17, and 24). Photosynthetic active radiation (PAR; *x*‐axis) is measured in μmol · photons · m^−2^ · s^−1^.
**Figure S9.** Measurements of the alpha, ETRmax and Ek for the RLCs run for *Posorira glacialis* (POR) and *Geminigera cryophila* (GEM) at the end of each light stage (LL, HL, and VLL).
**Figure S10.** Measurements of the quantum yields of PSII, Y(PSII), of the energy dissipated through non‐photochemical quenching, Y(NPQ), and of the energy dissipated through non‐regulated methods, Y(NO), over time (minutes) in the light stress induction and recovery experiments (LSREs) run for *Fragilariopsis cylindrus*. The left panels represent the variations of Y(PSII), whereas the middle and right panels exhibit the changes in the fractions of Y(PSII), Y(NPQ), and Y(NO) throughout the RLC for each species. Note that Y(PSII) in the left panel has been standardized to the initial Y(PSII) (*F*_v_/*F*_m_) and that Y(PSII) + Y(NPQ) + Y(NO) = 1. Gaps in the panels correspond to invalid measurements caused by very high variability.
**Table S1.** List of equations required to calculate all photo‐physiology indices calculated in the rapid light curves (RLCs) and the light stress induction‐recovery (LSREs; see main document).

## Data Availability

The data that support the findings of this study are available on request from the corresponding author.
