# Supplementary material for: Photoacclimation of cryptophytes and diatoms to light variability in the Western Antarctic Peninsula
Source: J Phycol. 2026 Apr 9;62(2):643–65. doi: 10.1111/jpy.70158 (PMC13103710; doi:10.1111/jpy.70158)
Supplement: Supplementary file 1 — Figure S1. Experimental light regime applied during the experiment. Photosynthetically active radiation (PAR; μmol · photons · m−2 · s−1) is shown as a function of time (days), with diel on:off cycles within each light stage. Shaded areas indicate the successive light treatments: low light (LL), high light (HL), very low light (VLL), followed by a return to HL and LL. Figure S2. Biovolume (μm3) variation for Geminigera cryophila (blue), Porosira glacialis (red), and Fragilariopsis cylindrus (yellow) throughout the treatment. Shaded areas in panels (a), (d), and (g) represent the periods of low and very low light. Figure S3. Variation of chlorophyll‐a concentration (Chl a) in percentage compared to the value measured on Day 7. Shaded areas in panels (a), (d), and (g) represent the periods under of the low (30–40 μmol · photons · m−2 · s−1) and very low (6 μmol · photons · m−2 · s−1) light stages. FRA, Fragilariopsis cylindrus; GEM, Geminigera cryophila; POR, Porosira glacialis. Figure S4. Agglomerative hierarchical clustering (Ward's method) of all samples collected during the treatment (N = 45) based on their intracellular pigment concentrations. * and° correspond to samples collected during high light phases (HL) and low light and very low light phases (LL + VLL), respectively. CRYP, Geminigera cryophila; FRAG, Fragilariopsis cylindrus; PORO, Porosira glacialis. Figure S5. Boxplot of pigment concentrations in each light phase (LL: low light, HL: high light, and VLL: very low light), showcasing the changes in pigments as part of photoacclimation for Geminigera cryophila, Porosira glacialis, and Fragilariopsis cylindrus. Each pigment's concentration was normalized between 0 and 1. Figure S6. Changes in F v/F m (maximum quantum yield of the PSII) throughout the experiment for each species (a) and the differences observed between different light phases (b). Data from all species were merged for the boxplots in (b). FRA, Fragilariopsis cylindrus; GEM, Geminigera cryophi [file JPY-62-643-s001.docx]

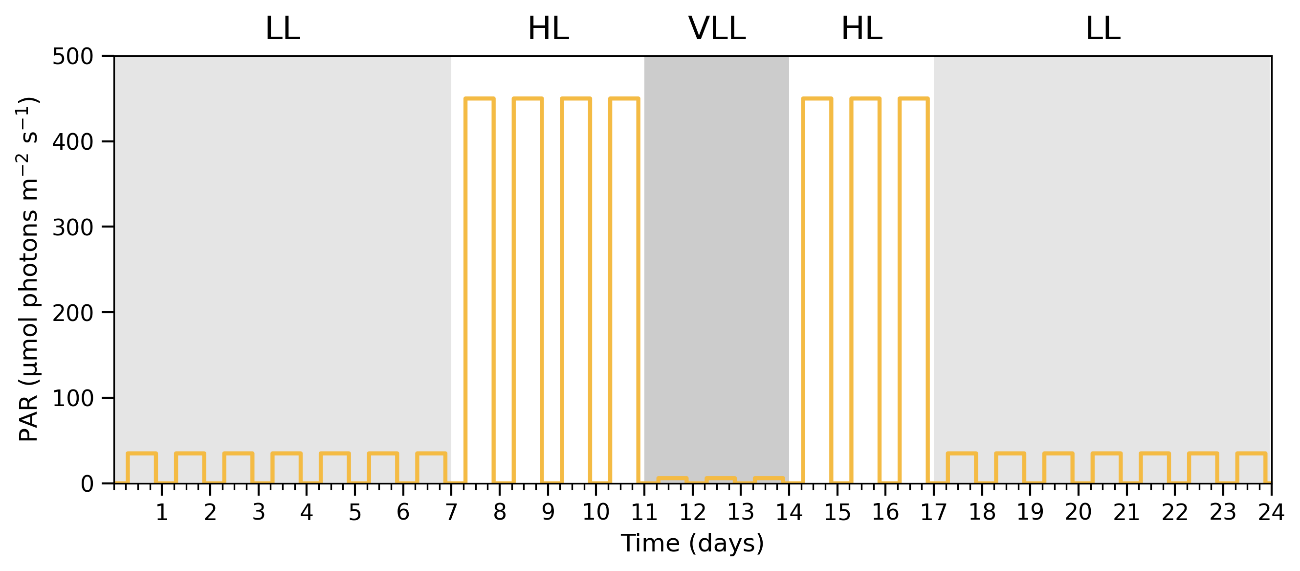


**Figure S1**: Experimental light regime applied during the experiment. Photosynthetically active radiation (PAR; µmol · photons · m^−2^ · s^−1^) is shown as a function of time (days), with diel on:off cycles within each light stage. Shaded areas indicate the successive light treatments: low light (LL), high light (HL), very low light (VLL), followed by a return to HL and LL.


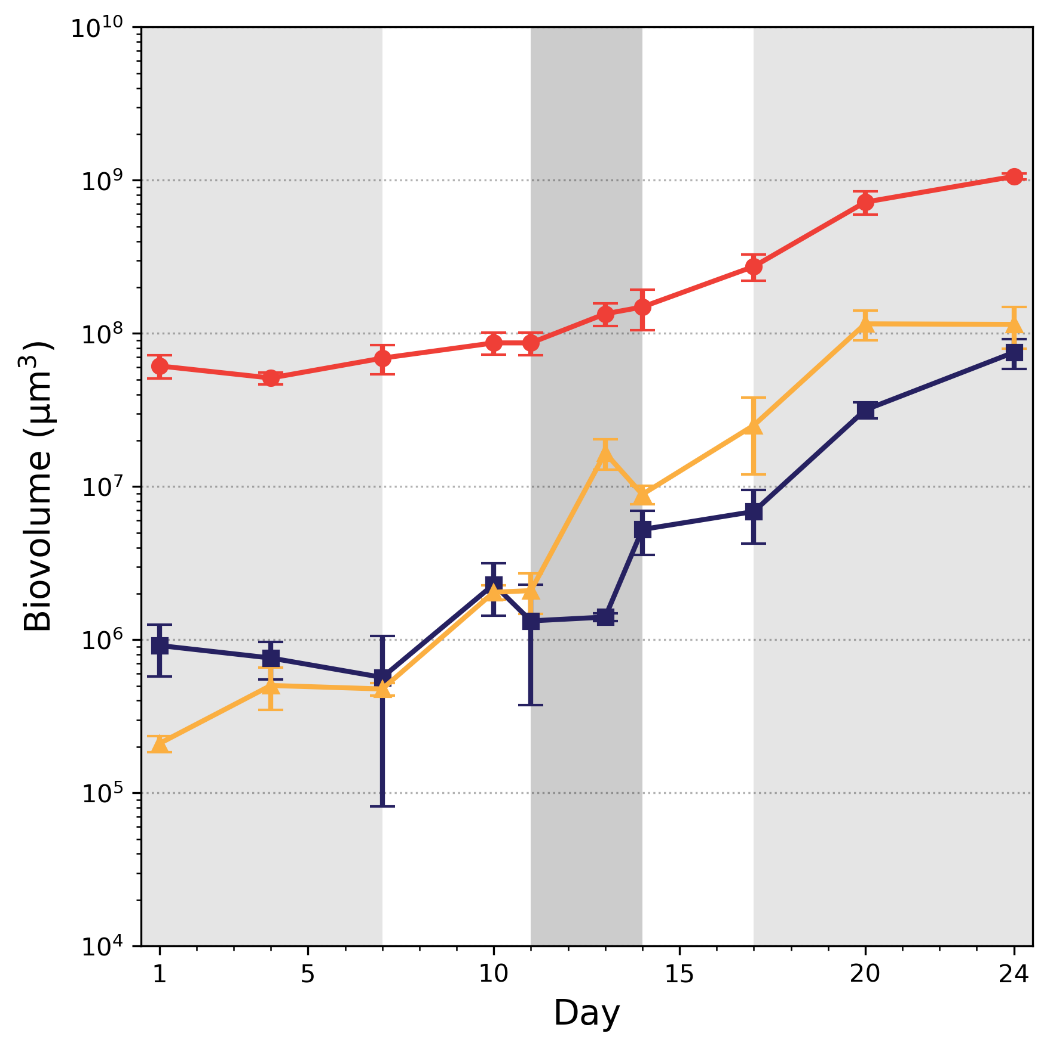


**Figure S2**: Biovolume (µm^3^) variation for *Geminigera* *cryophila* (blue), *Porosira* *glacialis* (red), and *Fragilariopsis* *cylindrus* (yellow) throughout the treatment. Shaded areas in panels a, d, and g represent the periods of low and very low light.


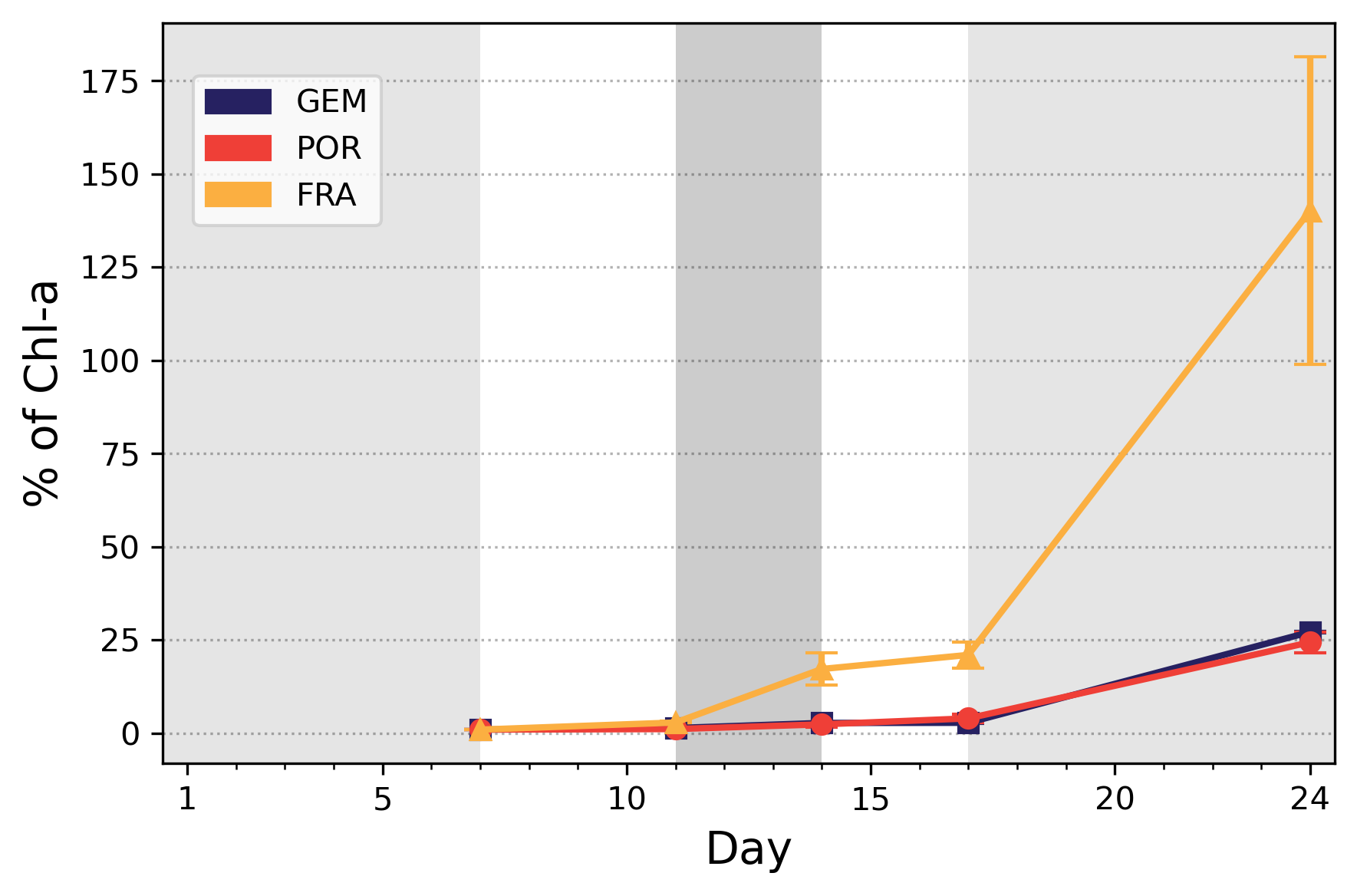


**Figure S3**: Variation of chlorophyll-*a* concentration (Chl *a*) in percentage compared to the value measured on Day 7. Shaded areas in panels a, d, and g represent the periods under of the low (30-40 µmol · photons · m^−2^ · s^−1^) and very low (6 µmol · photons · m^−2^ · s^−1^) light stages. GEM: *Geminigera cryophila*; POR: *Porosira glacialis*; FRA: *Fragilariopsis cylindrus*.


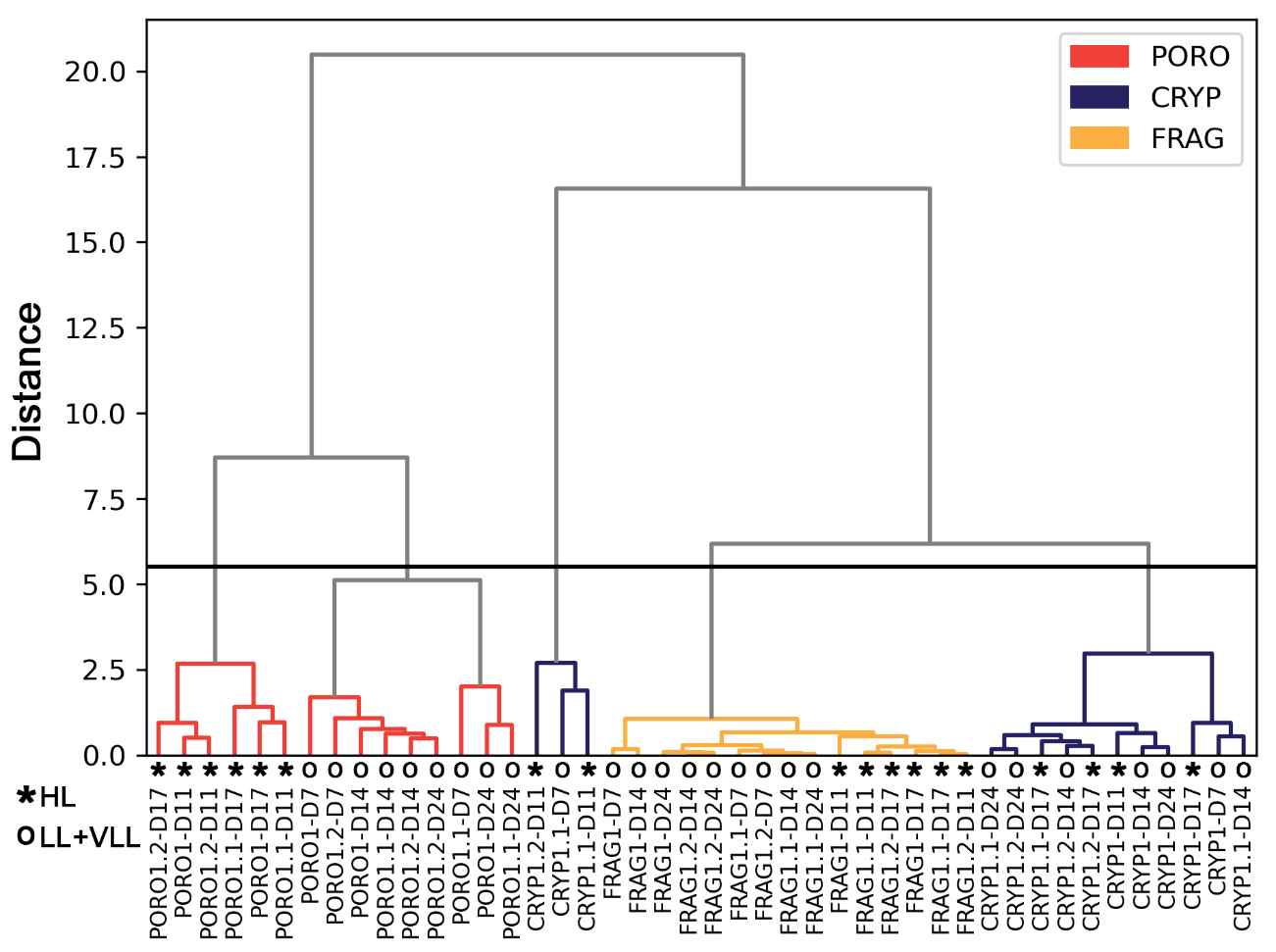


**Figure S4**: Agglomerative hierarchical clustering (Ward’s method) of all samples collected during the treatment (*N* = 45) based on their intracellular pigment concentrations. * and ° correspond to samples collected during high light phases (HL) and low light and very low light phases (LL + VLL), respectively. PORO: *Porosira* *glacialis*; CRYP: *Geminigera* *cryophila*; FRAG: *Fragilariopsis* *cylindrus*.


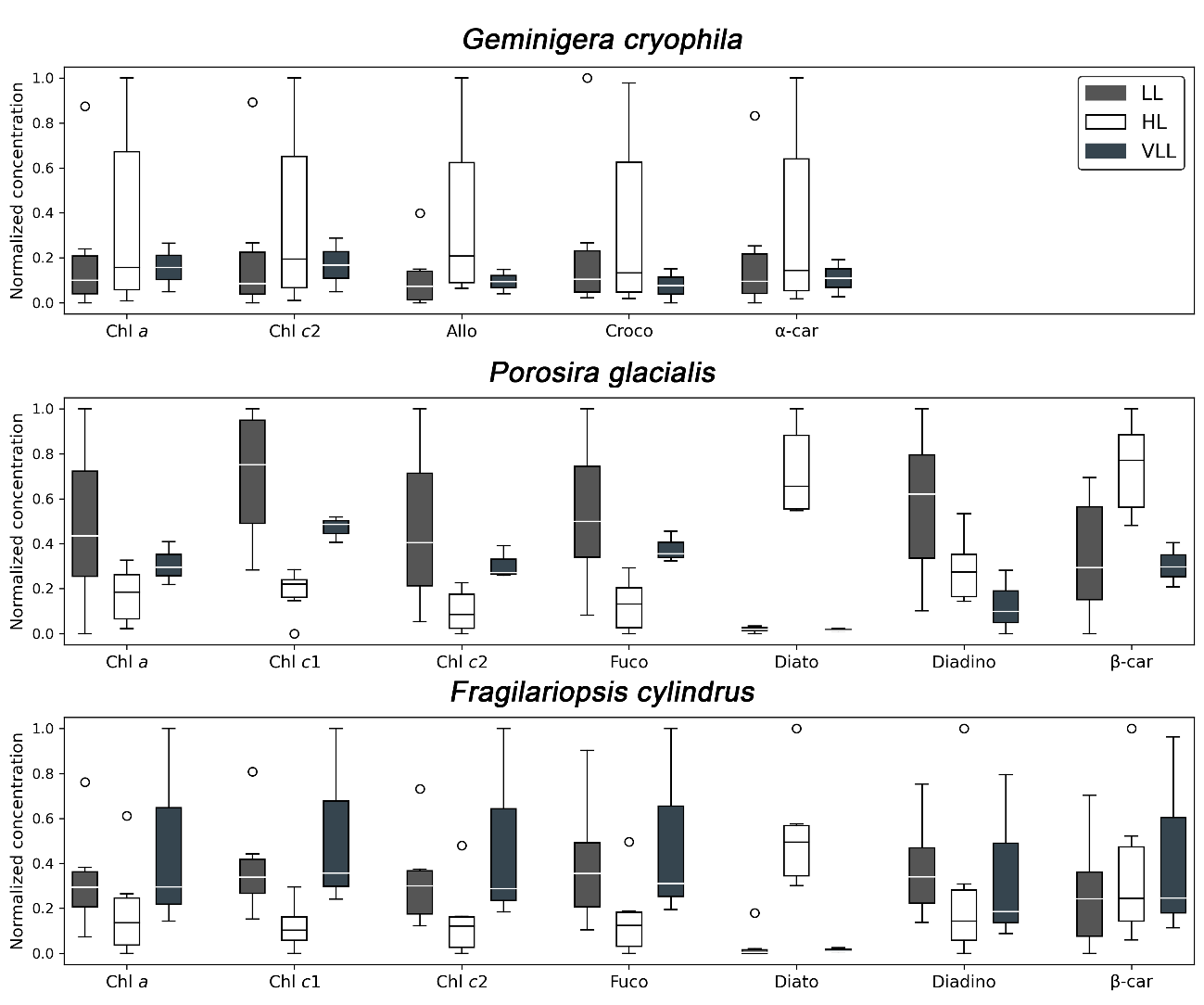


**Figure S5**: Boxplot of pigment concentrations in each light phase (LL: low light, HL: high light, and VLL: very low light), showcasing the changes in pigments as part of photoacclimation for *Geminigera cryophila*, *Porosira glacialis*, and *Fragilariopsis cylindrus*. Each pigment’s concentration was normalized between 0 and 1.


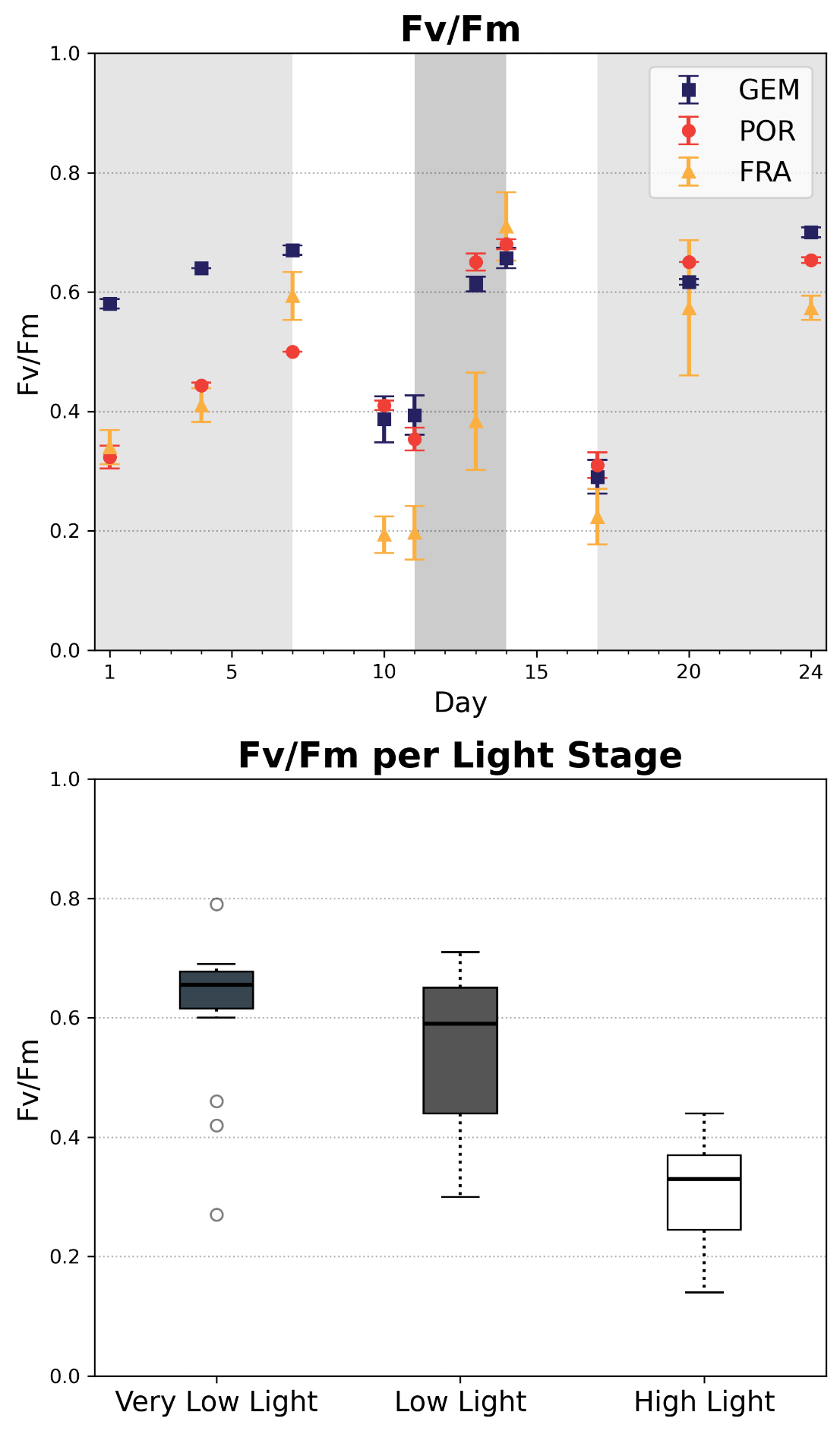


**Figure S6**: Changes in *F*v/*F*m (maximum quantum yield of the PSII) throughout the experiment for each species (a) and the differences observed between different light phases (b). Data from all species were merged for the boxplots in (b). GEM: Geminigera *cryophila*; POR: *Porosira* *glacialis*.; FRA: *Fragilariopsis* *cylindrus*.


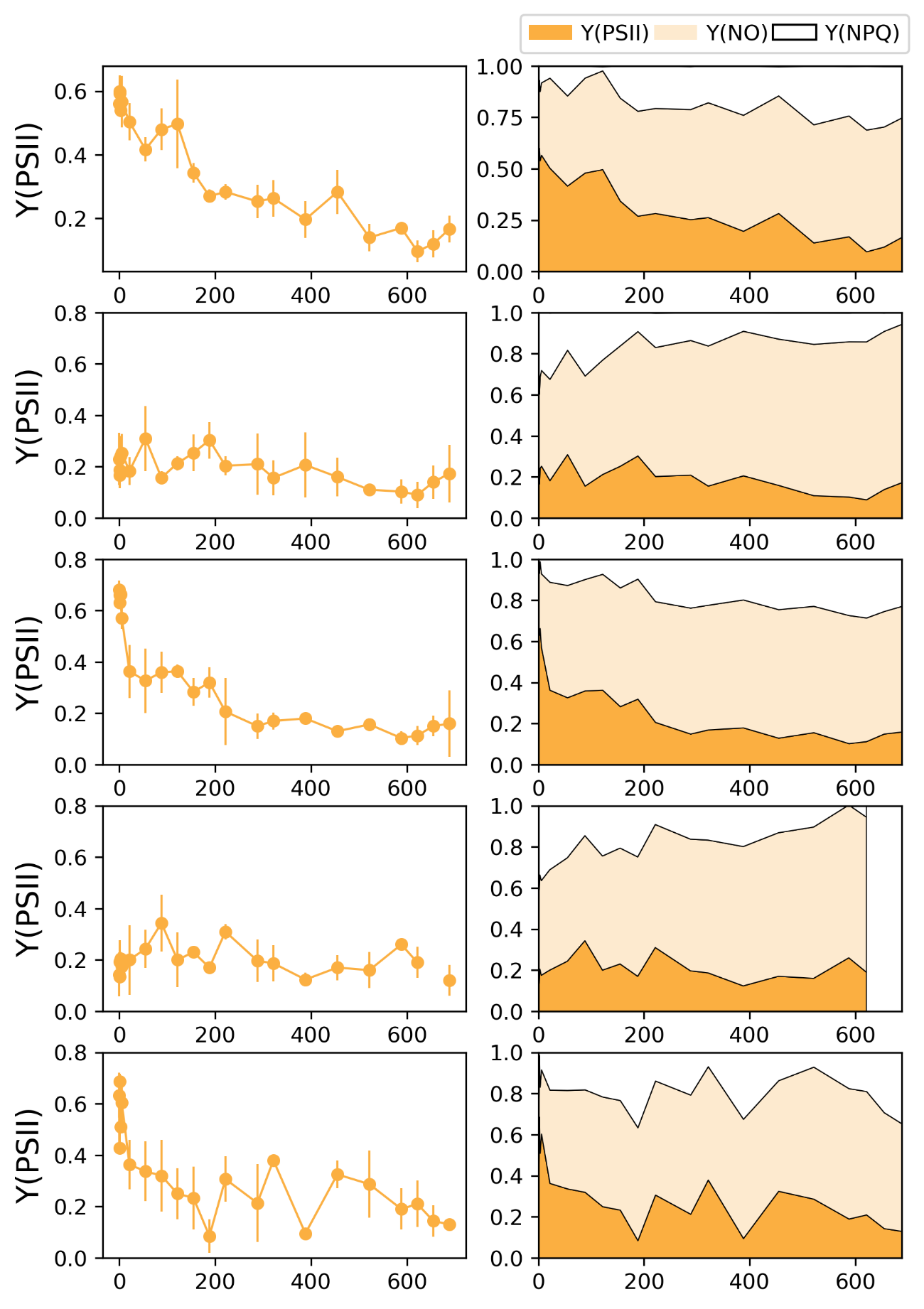


**Figure S7**: Measurements of the quantum yields of PSII, Y(PSII), of the energy dissipated through non-photochemical quenching, Y(NPQ), and of the energy dissipated through non-regulated methods, Y(NO), for each step of the rapid light curves (RLCs) run with *Fragilariopsis cylindrus*. The left panels represent the variations of Y(PSII), whereas the middle and right panels exhibit the changes in the fractions of Y(PSII), Y(NPQ), and Y(NO) throughout the RLC (note that Y[PSII] + Y[NPQ] + Y[NO] = 1). Gaps in the panels correspond to invalid measurements caused by very high variability.


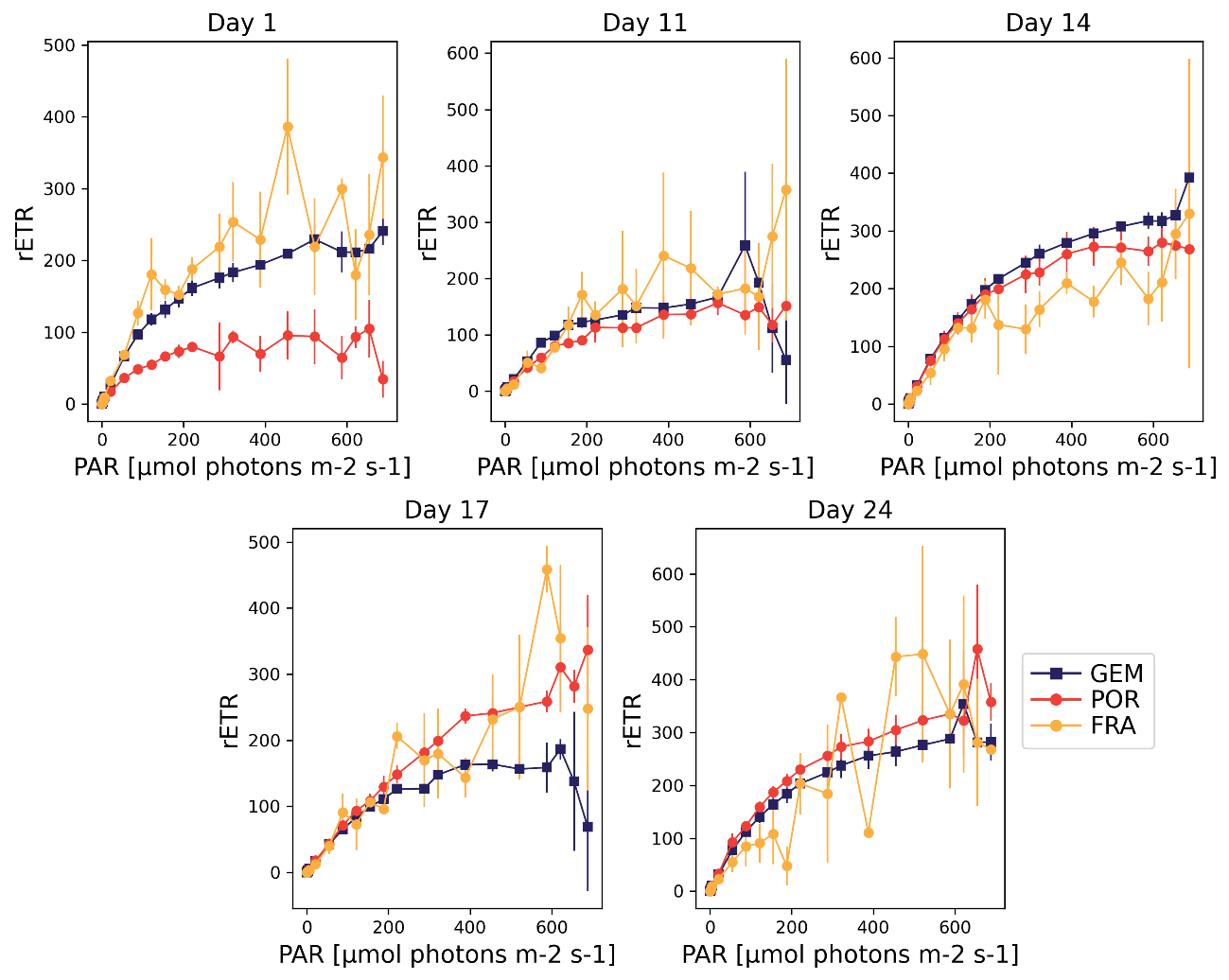


**Figure S8**: Measurements of the rETR (relative Electron Transport Rate) for each step of the rapid light curves (RLCs) run with all three species throughout the experiment (Days 7, 11, 14, 17, and 24). Photosynthetic active radiation (PAR; *x*-axis) is measured in µmol · photons · m^−2^ · s^−1^.


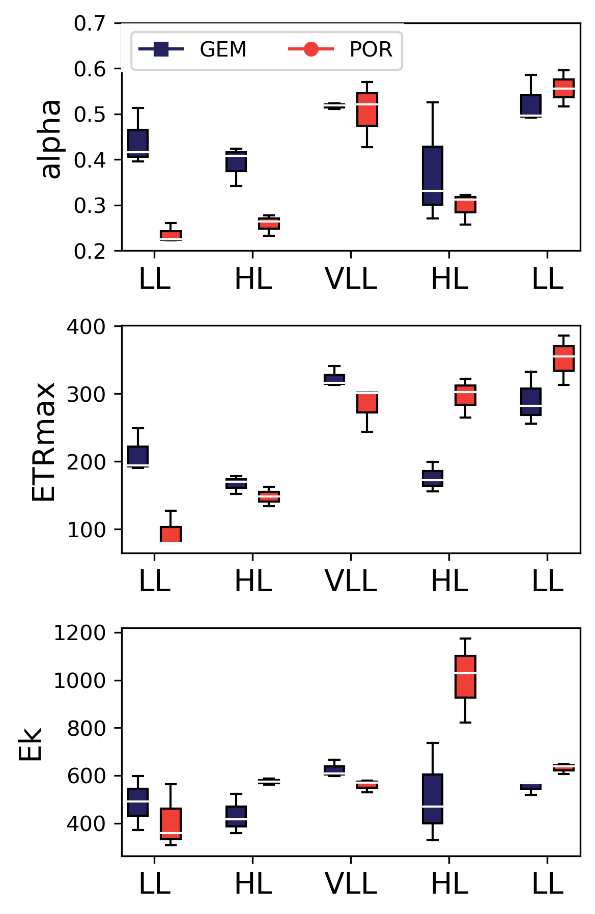


**Figure S9**: Measurements of the alpha, ETRmax and Ek for the RLCs run for *Posorira Glacialis* (POR) and *Geminigera cryophila* (GEM) at the end of each light stage (LL, HL, and VLL).


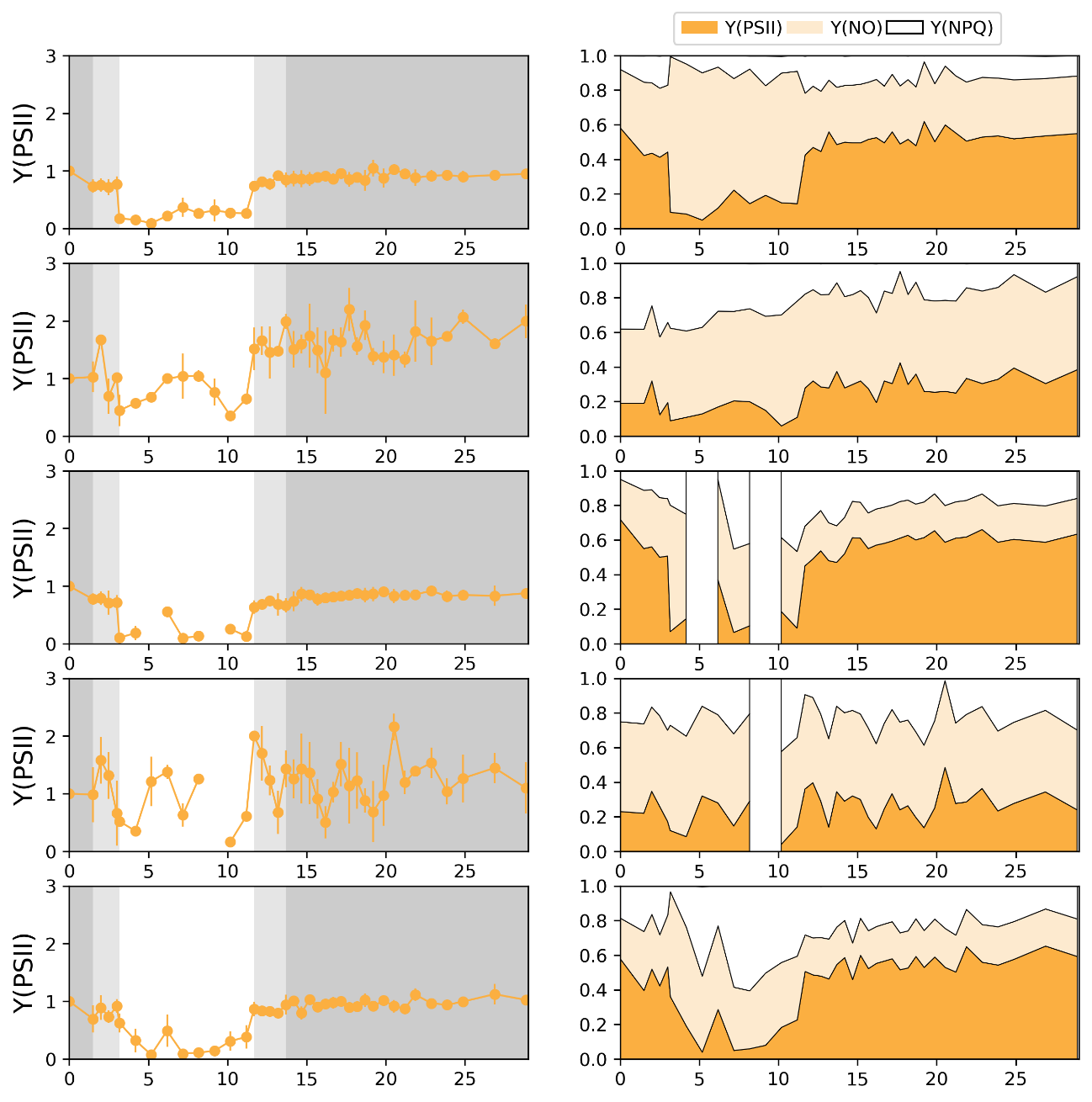


**Figure S10**: Measurements of the quantum yields of PSII, Y(PSII), of the energy dissipated through non-photochemical quenching, (Y(NPQ), and of the energy dissipated through non-regulated methods, Y(NO), over time (minutes) in the light stress induction and recovery experiments (LSREs) run for *Fragilariopsis cylindrus*. The left panels represent the variations of Y(PSII), whereas the middle and right panels exhibit the changes in the fractions of Y(PSII), Y(NPQ), and Y(NO) throughout the RLC for each species. Note that Y(PSII) in the left panel has been standardized to the initial Y(PSII) (Fv/Fm) and that Y(PSII) + Y(NPQ) + Y(NO) = 1. Gaps in the panels correspond to invalid measurements caused by very high variability.

**Table S1**: List of equations required to calculate all photo-physiology indices calculated in the rapid light curves (RLCs) and the light stress induction-recovery (LSREs; see main document). rETRmax was based on the model proposed by Platt et al., 1980^1^.

| **Parameter calculated** | **Equation** |
| --- | --- |
| Y(PSII) | $Y\left( \mathrm{PSII} \right)=\frac{Fm^{'}-F}{Fm^{'}}$ |
| Y(NPQ) | $Y\left( \mathrm{NPQ} \right)=\frac{F}{Fm^{'}}-\frac{F}{Fm}$ |
| Y(NO) | $Y\left( \mathrm{NO} \right)=\frac{F}{Fm}$ |
| rETR | $rETR=Y\left( \mathrm{PSII} \right) \times E$ |
| rETR_max_ | $\mathrm{rETR}_{\max}=P_{s}\left( \frac{\alpha}{\left[ \alpha+\beta\right]} \right)\left( \frac{\beta}{\left[ \alpha+\beta\right)} \right)^{\frac{\beta}{\alpha}}$ |
| *E*_k_ | $E_{k}=\frac{\mathrm{ETR}_{\max}}{\alpha}$ |
| Total absorbed excitation energy in PSII (1) | 1 = Y(PSII) + Y(NPQ) + Y(NO) |

^1^Platt, T., Gallegos, C. L., & Harrison, W. G. (1980). Photoinhibition of photosynthesis in natural assemblages of marine phytoplankton. *Journal of Marine Research,* 38(4), 687–701.

*F*: Fluorescence yield measured briefly before application of a Saturation Pulse.

*F*m: Maximal fluorescence yield of dark-adapted sample with all PS II centres closed.

*F*m': Maximal fluorescence yield of illuminated sample with all PS II centres closed.

Y(PSII): Quantum yield of photochemical energy conversion in PS II.

Y(NPQ): Quantum yield of regulated non-photochemical energy loss in PS II.

Y(NO): Quantum yield of non-regulated non-photochemical energy loss in PS II.

rETR: Electron transport rate.

rETR_max_: Maximum electron transport rate.

P_s_ = 2000

ɑ = initial slope of RLC.

β: parameter that characterized the downturn of the curve after reaching rETR_max_

*E*k: Minimum saturation irradiance.
